# Use of gonadotropin-releasing hormone agonists in transgender and gender diverse youth: a systematic review

**DOI:** 10.3389/fendo.2025.1555186

**Published:** 2025-05-14

**Authors:** Gianluca Tornese, Raffaella Di Mase, Jessica Munarin, Silvia Ciancia, Fabiana Santamaria, Daniela Fava, Egidio Candela, Donatella Capalbo, Carla Ungaro, Nicola Improda, Pierluigi Diana, Patrizia Matarazzo, Laura Guazzarotti, Tommaso Toschetti, Vanessa Sambati, Gianluca Tamaro, Giulia Bresciani, Maria Rosaria Licenziati, Maria Elisabeth Street, Tommaso Aversa, Maurizio Delvecchio, Maria Felicia Faienza, Lorenzo Iughetti, Valeria Calcaterra, Luisa de Sanctis, Mariacarolina Salerno, Roberto Franceschi

**Affiliations:** ^1^ Institute for Maternal and Child Health IRCCS “Burlo Garofolo”, Trieste, Italy; ^2^ Department of Medicine, Surgery and Health Sciences, University of Trieste, Trieste, Italy; ^3^ Pediatric Endocrinology Unit, Department of Mother and Child, University Hospital Federico II, Naples, Italy; ^4^ Regina Margherita Children’s Hospital, Pediatric Endocrinology, Department of Public Health and Pediatric Sciences, University of Turin, Turin, Italy; ^5^ Department of Internal Medicine and Pediatrics, Ghent University, Ghent, Belgium; ^6^ Department of Neuroscience, Rehabilitation, Ophthalmology, Genetics, Maternal and Child Health, University of Genoa, Genoa, Italy; ^7^ Pediatric Endocrinology Unit, Department of Pediatrics, IRCCS Istituto Giannina Gaslini, Genoa, Italy; ^8^ Pediatric Unit, IRCCS Azienda Ospedaliero-Universitaria di Bologna, Bologna, Italy; ^9^ Department of Medical and Surgical Sciences, Alma Mater Studiorum, University of Bologna, Bologna, Italy; ^10^ Pediatric Endocrinology Unit, Department of Translational Medical Sciences, University of Naples Federico II, Naples, Italy; ^11^ Maternal and Child Unit, Local Health Unit, ASL Napoli 1 Centro, Naples, Italy; ^12^ Neuro-Endocrine Diseases and Obesity Unit, Department of Neurosciences, Santobono-Pausilipon Children’s Hospital, Naples, Italy; ^13^ Department of Medicine and Surgery, University of Parma, Parma, Italy; ^14^ Endocrinology Unit, Pediatric Department, University of Padua, Padua, Italy; ^15^ Pediatric Clinic, Pietro Barilla Children’s Hospital, Department of Medicine and Surgery, University Hospital of Parma, Parma, Italy; ^16^ Department of Human Pathology of Adulthood and Childhood, University of Messina, Messina, Italy; ^17^ Department of Biotechnological and Applied Clinical Sciences, University of L’Aquila, L’Aquila, Italy; ^18^ Pediatric Unit, Department of Precision and Regenerative Medicine and Ionian Area, University of Bari “A. Moro”, Bari, Italy; ^19^ University of Modena and Reggio Emilia, Modena, Italy; ^20^ Pediatrics Unit, University Hospital of Modena, Modena, Italy; ^21^ Pediatric and Adolescent Unit, Department of Internal Medicine, University of Pavia, Pavia, Italy; ^22^ Pediatric Department, Buzzi Children’s Hospital, Milan, Italy; ^23^ Department of Pediatrics, Santa Chiara Hospital of Trento, Azienda Provinciale per i Servizi Sanitari della Provincia Autonoma di Trento, Trento, Italy; ^24^ Centre for Medical Sciences, University of Trento, Trento, Italy

**Keywords:** gender dysphoria (GD), gender incongruence, adolescence, GnRH analog (GnRHa), LHRH analog, transgender and gender diverse (TGD), transgender and gender diverse youth

## Abstract

**Introduction:**

Puberty suppression using gonadotropin-releasing hormone agonists (GnRHa) is a reversible medical intervention that halts endogenous puberty, allowing transgender and gender-diverse (TGD) adolescents to avoid the development of secondary sexual characteristics that may cause psychological distress. This pause in pubertal progression provides time to explore gender identity or facilitates alignment with affirmed gender in those with an established identity. While widely used, long-term evidence on the efficacy and safety of GnRHa in this population remains limited. This systematic review aims to synthesize current data on the benefits and potential risks of GnRHa in TGD adolescents.

**Methods:**

We conducted a comprehensive literature search across PubMed, EMBASE, Cochrane Library, and other databases, covering studies published from February 2011 to February 2024. Eligible studies included adolescents under 18 with gender dysphoria or incongruence treated with GnRHa, reporting outcomes related to efficacy or side effects. Fifty-one studies met inclusion criteria, and data on physical health, mental health, bone density, fertility, and adverse events were extracted and assessed using the GRADE approach.

**Results:**

Of the 51 studies, 22 were rated as moderate to high-quality evidence. GnRHa effectively suppressed puberty and secondary sex characteristics. Effects on growth and body composition varied; bone mineral density declined during treatment, particularly in AMAB individuals. Mental health improved significantly, including reduced depression, anxiety, and suicidality—especially when GnRHa was followed by gender-affirming hormone therapy (GAHT). Quality of life improved over time, while body dissatisfaction often persisted during suppression and improved after GAHT or surgery. No moderate- or high-quality evidence was found on fertility, sexual function, or cancer risk.

**Conclusion:**

GnRHa is effective in halting puberty and improving mental health in TGD adolescents. However, key clinical and ethical considerations—such as bone health monitoring, fertility counseling, psychological support, and informed decision-making—must guide treatment. Long-term safety remains uncertain, particularly regarding skeletal health, reproductive outcomes and cancer risk. A precision medicine approach and co-produced longitudinal studies are essential to support safe, individualized care.

**Systematic review registration:**

https://www.crd.york.ac.uk, identifier CRD42024528334.

## Introduction

Transgender and gender diverse (TGD) individuals are those whose gender identity differs from the sex assigned to them at birth. Within this population, two clinical concepts are commonly used to describe experiences related to gender identity: gender incongruence (GI) and gender dysphoria (GD). GI, as defined in the International Classification of Diseases (ICD-11) (1), refers to a persistent incongruence between a person’s experienced gender and their assigned gender, without necessarily involving distress. Conversely, GD, as outlined in the Fifth Edition of the Diagnostic and Statistical Manual of Mental Disorders (DSM-5) (2), is characterized by clinically significant distress or discomfort resulting from this incongruence.

When GD or GI persist or emerge during puberty, TGD adolescents may be eligible for puberty suppression using gonadotropin-releasing hormone analogs (GnRHa) (3). This intervention serves multiple purposes: it provides time for individuals to explore their gender identity, prevents the development of irreversible secondary sex characteristics that may cause distress, and facilitates a smoother transition to gender-affirming hormone therapy (GAHT) if pursued (4). Conversely, GAHT leads to partially irreversible changes in secondary sex characteristics, such as breast development or deepened voice and facial hair (3). Preventing puberty that is incongruent with gender identity is particularly important, as it can positively affect mental health outcomes, and reduce the need for later invasive and expensive surgeries, such as chest surgery or facial feminization surgery (5).

Despite increasing interest in the effects of GnRHa treatment in TGD adolescents, significant gaps remain in the literature. In 2018 Chew et al. (6) conducted a systematic review concluding that low-quality evidence suggests hormonal treatments achieve their intended physical effect; however, evidence regarding psychological and cognitive impact remained insufficient. In 2023 Ludvigsson et al. performed a systematic review regarding hormone treatment in TGD adolescents, analyzing studies published until November 2021 (7). They found no randomized controlled trials and noted that few longitudinal observational studies were limited by small sample sizes. Consequently, they concluded that the long-term effects of hormone therapy on psychosocial health could not be evaluated.

A more recent systematic review synthesized studies assessing puberty suppression outcomes in TGD adolescents (8). The review included 50 studies and found consistent moderate-quality evidence demonstrating the efficacy of GnRHa in reducing gonadotropin and sex steroid levels, with the effects on secondary sex characteristics varying depending on whether treatment is initiated in early or mid-puberty. However, it raised significant concerns regarding bone mineral density (BMD) and linear growth during treatment, with studies reporting reductions in BMD and height increases that did not align with expected growth trajectories. Importantly, the review noted a lack of high-quality research assessing the impact of GnRHa on GD, mental health, psychosocial health, cognitive development, and fertility outcomes.

To address these gaps, this systematic review provides an updated synthesis of evidence on the efficacy and safety of GnRHa in TGD adolescents, with a focus on studies of moderate to high quality, assessed using the GRADE approach. By examining key outcomes—including physical development, mental health, and bone health—this review aims to contribute to a more comprehensive understanding of the role of GnRHa in gender-affirming care, while also identifying areas where further research is needed.

## Materials and methods

The protocol of this systematic review was registered in PROSPERO on March 24, 2024, with registration number CRD42024528334.

### Search strategy

We searched electronic databases (Pubmed, EMBASE, The Cochrane Library, Web of Science, Clinicaltrial.gov, International Clinical Trials Registry Platform) for studies published between February 1, 2011 (the date the first study was published) and February 1, 2024. Search terms, or “MESH” (MEdical Subject Headings) for this systematic review included different combinations:

“Gender Dysphor*” or “Gender incongruen*” or transgender or nonbinary

AND “Gonadotropin-releasing hormone agonist” or “GnRH Analogue” AND “puberty block*” or “puberty suppress*” or “puberty inhibit*”. The exact terms used to search each database are reported in **Supplementary Material S1**.

We also screened the reference list of eligible studies to avoid missing any relevant studies. We used the “free-text search” technique for fertility, sexual function, side effects, and risk of cancer outcomes, to improve the performance of our search, and more documents that matched the search criteria, not selected by the electronic databases, were included.

### Criteria for study selection

To minimize the risk of selection bias, two Authors (RF, GTo) independently conducted a systematic literature search according to the PICOS model (Population, Intervention, Comparison, Results, Study design). We formulated five questions related to the GnRHa’s efficacy, and for each one, we established the outcomes listed in **Table 1**:

**Table 1:** PICOS (Population, Intervention, Comparison, Results and Study design) framework for the systematic review.

Population	Adolescents (<18 years, Tanner Stage ≥ 2) with GD/GI
Intervention	Puberty suppression with or without GAHT
Comparison	Adolescents with GD/GI not treated or age-matched individuals
Outcomes	Q1) PHYSICAL CHANGES AND HORMONE LEVELS: pubertal stage, anthropometry (height velocity, final height), body composition (% fat mass, % lean mass), secondary sexual characteristics, blood pressure; % of TGD adolescents that proceed to GAHT, voice, breast, menstrual bleeding, hormone levels. Q2) BONE HEALTH: bone age, bone mineral density, bone turnover, bone geometry. Q3) MENTAL HEALTH: psychological well-being, QoL, social life, satisfaction, body image, depression-anxiety, self-harm and suicidal ideation, intelligence quotient (IQ), autism. Q4) FERTILITY AND SEXUAL FUNCTION: data on fertility and satisfaction with sexual function. Q5) SIDE EFFECTS AND RISK OF CANCER: physical, hematological, biochemical, cardiovascular, % of persistence of dysphoria. Prostate cancer, breast cancer, mortality.
Study design	Randomized controlled trials (RCTs), observational studies (cohort, case-control), exploratory studies, and a mix of qualitative and quantitative studies.

Inclusion criteria were: i) study population: adolescents (age <18 years, Tanner Stage ≥ 2) who had GD/GI treated with GnRHa, and their follow-up data were reported; ii) study type: observational studies (cohort, case-control), exploratory studies (that consist in additional preliminary research to determine priorities and problems to be solved), a mix of qualitative (data from surveys, interviews) and quantitative studies; iii) review articles were excluded, but their reference lists were screened to identify potentially eligible studies; iv) only full papers were included, whereas conference abstracts were not included; v) data on the modality and efficacy of the hormonal therapy (puberty suppression with GnRHa, with or without GAHT), and TGD adolescents’ characteristics: age at the start of therapy, pubertal status, duration of treatment, outcomes, rate of discontinuation and side effects; vi) publication date: last 15 years (2011-2024). Exclusion criteria: i) data available only for adults ≥18 years without baseline status (cross-sectional studies); ii) data only at baseline, without follow-up; iii) non-human animal data; iv) case reports; studies with <10 patients who underwent GnRHa therapy; v) full paper not available; vi) study not yet published; vii) studies not reporting the selected outcomes; viii) studies on GAHT without data on GnRHa treatment; studies on Gender Affirming Surgery (GAS).

Case reports were excluded from the review due to their high potential for bias—both in terms of publication and their focus on rare or unusual cases—as well as their limited generalizability.

### Data extraction and management

The same two independent investigators (RF, GTo) screened for inclusion in the title and abstract of all the studies identified by the search strategy. Any discrepancies between them were resolved by consensus. After abstract selection, 25 investigators were subdivided into the five research questions (Q1 to Q5) and conducted the full-text review.

The following characteristics were evaluated for each study in the full paper: i) reference details: authorship(s); published or unpublished; year of publication; period in which the study was conducted; other relevant cited papers; ii) study characteristics: study design, topic, treatment period, follow up duration, region; iii) population characteristics: number of participants (assigned male at birth, AMAB; assigned female at birth, AFAB) who underwent puberty suppression or GAHT, age and demographic data; comparator characteristics; iv) methodology: measures to assess the outcomes; v) main results: outcome measures. Data extraction was completed in duplicate.

### Assessment of the certainty of the evidence

We used the GRADE approach to rank the quality of evidence (www.gradeworkinggroup.org) for the included studies. Two authors (RF, GTo) independently assessed the certainty of the evidence for each of the outcomes, and any differences between them were resolved by consensus. The two authors used ROBINS-I (“Risk Of Bias In Non-Randomized Studies - of Interventions”) as a tool to ensure consistency and accuracy (9). In the case of risk bias in the study design, imprecision of estimates, inconsistency across studies, indirectness of the evidence, and publication bias, the recommended option of decreasing the level of certainty by one or two levels according to the GRADE guidelines was applied. Using the ROBINS-I tool, the GRADE framework assigns an initial certainty rating of high for outcome data from RCTs and low for observational studies. Certainty can be downgraded based on five domains: limitations in study design and execution (risk of bias), inconsistency (heterogeneity), indirectness (PICO and applicability), imprecision, and publication bias. Conversely, three factors can increase certainty: a large magnitude of effect, opposing plausible residual bias or confounding, and a dose-response gradient. We have reported the cut-off values (median values) used to downgrade the level of certainty by one or two levels.

The GRADE approach results in an assessment of the certainty of a body of evidence and allocation to one of four grades, reported in **Table 2**:

**Table 2:** GRADE classification of evidence certainty levels.

High	Further research is very unlikely to change confidence in the estimate of the effect
Moderate	Further research is likely to have an important impact on confidence in the estimate of the effect and may change the estimate
Low	Further research is very likely to have an important impact
Very low	Any estimate of the effect is very uncertain.

### Data synthesis

Starting from Tables that reported the details of each study (**Supplementary Tables**), relevant information reported in the ones with a moderate-high quality level of evidence, were categorized into separate “tables of evidence”, one for each outcome (**Tables 3A-C**).

**Table 3A:** Summary of findings on physical changes and hormone levels on GnRHa treatment in TGD adolescents.

Reference	Study design/ Population	Intervention (duration)	Height	Sexual characteristics	Gonadotropins and sexual steroids	Body composition	Lipids /glucose
Boogers 2022 (10)	R/161 (161 AMAB, 0 AFAB)	GnRHa (2.4 ± 0.8 y) GAHT	↓ (3m) during GnRH ↓ (adult) High dose E and EE ↓ Bone age during GnRH	N/A	N/A	N/A	N/A
Fisher 2024 (4)	P/36 (14 AMAB, 22 AFAB)	GnRHa (3-12 m)	↓ HT centile in AMAB	↓ Tanner stage	↓ FSH/LH and T in AMAB/E in AFAB	↑ BMI in AFAB	↓ HDL in AMAB =
Klaver 2018 (11)	R/192 (71 AMAB, 121 AFAB)	GnRHa AMAB: 2.1 y (1.0-2.8) AFAB: 1.0 years (0.5-2.9) GAHT	N/A	N/A	N/A	WHR and body composition changed toward the affirmed sex	N/A
Klaver 2020 (12)	R/192 (71 AMAB, 121 AFAB)	GnRHa AMAB: 2.1 y (1.0-2.8); AFAB: 1.0 y (0.5-2.9) GAHT	N/A	N/A	N/A	> % of obesity vs controls	= Changes in lipids, insulin, HOMA IR, BP and BMI similar to controls
Navabi 2021 (13)	R/172 (51 AMAB, 119 AFAB)	GnRHa (N/A)	N/A	N/A	N/A	↑ total body fat % increase in gynoid (%fat), and android (%fat) in AFAB and AMAB was detected without changes in BMI *z* score	N/A
Schagen 2016 (14)	P/116 (49 AMAB, 67 AFAB)	GnRHa (N/A)	↓ (3m) during GnRH	↓ (3m) during GnRH	↓ (3m) during GnRH	↓ Lean body mass % and ↑ fat at 1y	N/A
Schagen 2018 (15)	P/127 (73 AMAB, 54 AFAB)	GnRHa (2y) GAHT	N/A	N/A	↑ DHEAS / ↑ A	N/A	N/A
Schulmeister 2022 (16)	P/55 (26 AMAB, 29 AFAB)	GnRHa (1y)	No differences vs controls in early PS Lower HV in later Tanner stage PS initiation	N/A	N/A	N/A	N/A
Valentine 2022 (17)	R-C/4172 (1407 AMAB, 2766 AFAB)	GnRHa (N/A) GAHT	N/A	N/A	N/A	↑ overweight/obesity	↑ dyslipidemia, liver dysfunction and hypertension
Van de Grift 2020 (18)	R/200 (66 AMAB, 134 AFAB)	GnRHa (3y) GAHT	No difference in FH among early, later, or no GnRHa	↓ during GnRH	N/A	No difference in BMI SDS among early, later, or no GnRHa	N/A
Willemsen 2023 (19)	R/146 (0 AMAB, 146 AFAB)	GnRHa (3.1±0.9 y) + Testosteron	↓ HV (during GnRHa) ↑ HV (during GAHT) FH exceeded PAH by 3.0 ± 3.6 cm and MPH by 3.9 ± 6.0 cm	N/A	N/A	N/A	N/A

A, androstenedione; AFAB, assigned female at birth; AMAB, assigned male at birth; AST, aspartate aminotransferase; BMI, body mass index; BMIz, BMI z-score; BP, blood pressure; C, cross-sectional; DBP, diastolic blood pressure; E, estrogen; EE, ethinyl estradiol; FSH, follicular hormone; f/up, follow-up; FH, final height; GAHT, gender-affirming hormone therapy; GD, gender dysphoria; GnRHa, gonadotropin-releasing hormone agonist; HbA1c, hemoglobin A1C; HDL, high-density lipoprotein; HOMA-IR, homeostatic model assessment for insulin resistance; HT, height; HTz, height z-score; im, intramuscularly; LBW, lean body weight; LDL, low-density lipoprotein; LH, luteinizing hormone; N/A, not available; NS, not significant; P, prospective; PAH, predicted adult height; pc, percentile; PS, pubertal suppression; R, retrospective; SBP, systolic blood pressure; sc, subcutaneously; T, testosterone; TBF, total body fat; TGD, transgender and gender-diverse; TV, testicular volume; WT, weight; WTz, weight z-score; WHR, waist-hip ratio; y, year(s).

Only studies with moderate-high level quality of evidence are included.Arrow down: reduced; Arrow up: increased.

**Table 3B:** Summary of findings on bone outcomes of GnRHa treatment in TGD adolescents. Only studies with moderate-high level quality of evidence are included.

Reference	Design/Population	Intervention (duration)	Bone mineral density	Bone turnover
Carmichael 2021 (20)	P/44 (25 AMAB, 19 AFAB)	GnRHa (29 m AFAB - 37 m AMAB)	No change from baseline in LS BMD at 12 m nor in hip BMD at 24 and 36 m	N/A
Schagen 2020 (21)	P/121 (51 AMAB, 70 AFAB)	GnRHa (1.8 AFAB - 2.0 AMAB) + GAHT (3 y)	BMAD =/ ↓ during GnRHa ↑ BMAD during GAHT Z-scores normalized in AFAB but remained <0 in AMAB	P1NP↓/=, P3NP↓, osteocalcin↓, 1CTP↓ After 3 y of GAHT: P1NP↓, P3NP↓/=, osteocalcin↓, 1CTP↓
Van der Loos 2023 (22)	R/75 (25 AMAB, 50 AFAB)	GnRHa (1.5 y both AFAB and AMAB) + GAHT (5.7 y AFAB - 5.3 y AMAB)	BMD Z-scores returned to pretreatment levels except LS Z-scores in AMAB	N/A
Vlot 2017 (23)	R/70 (28 AMAB, 42 AFAB)	GnRHa (1.5 y AFAB – 1.3 y AMAB) + GAHT (5.4 y AFAB – 5.8 y AMAB)	BMD Z-scores ↓ during GnRHa ↑after 24 m of GAHT	↓P1NP and 1CTP during GnRHa

1CTP, cross-linked telopeptide of type 1 collagen; AFAB, assigned female at birth; AMAB, assigned male at birth; BMAD, bone mineral apparent density; BMD, bone mineral density; GAHT, gender-affirming hormone therapy; GnRHa, gonadotropin-releasing hormone analogues; LS, lumbar spine; m, months; N/A, not available; P, prospective; P1NP: procollagen type 1 N propeptide; P3NP, procollagen type 3 N propeptide; R, retrospective; TGD, transgender and gender-diverse; y, years.Arrow down: reduced; Arrow up: increased.

**Table 3C:** Summary of findings on mental health outcomes of GnRHa treatment in TGD adolescents. Only studies with moderate-high level quality of evidence are reported.

Reference	Design/Population	Intervention (duration)	Global function/Cognition behavioral and emotional problems	Suicide ideation	Depression/Anxiety	QoL/wellbeing	Body Dissatisfaction
Achille 2020 (24)	P/50 (17 AMAB, 33 AFAB)	GnRHa (18 m) and/or GAHT	N/A	↓	↓	↑ QoL	N/A
Arnoldussen 2022 (25)	R/72 (27 AMAB, 45 AFAB)	GnRHa (2,4 y) and/or GAHT	The IQ scores and educational achievement were not significantly different between AMAB and AFAB	N/A	N/A	N/A	N/A
Costa 2015 (26)	P/201 (77 AMAB; 124 AFAB)	GnRHa (18m) + psychological support	GD adolescents receiving also GnRHa had > psychosocial functioning (+12m)	N/A	N/A	↑ well-being	N/A
De Vries 2011 (27)	P/70 (33 AMAB, 37 AFAB)	GnRHa (2 y)	↓behavioral and emotional problems, depressive symptoms ↑ psychological functioning	N/A	↓/=	N/A	= Body image and gender dysphoria (> natal female)
De Vries 2014 (28)	P/55 (22 AMAB, 33 AFAB)	GnRHa (N/A)	↑ psychological functioning	N/A	↓	↑ QoL	**↓**
Fisher 2024 (4)	P/36 (14 AMAB, 22 AFAB)	GnRHa (3-12 m)	↑ psychological functioning	↓	↓	N/A	**↓**
Tordoff 2022 (29)	P/104 (27 AMAB, 63 AFAB, 10 NB, 4 unknown)	GnRHa (N/A) + GAHT	N/A	↓ (PS and GAH)	↓ (PS and GAH)	N/A	N/A
Van der Miesen 2020 (30)	P/178 (68 AMAB, 110 AFAB)	GnRHa (N/A) vs no treatment	N/A	↓	↓	N/A	N/A

AFAB, assigned female at birth; AMAB, assigned male at birth; NB, non-binary; P, prospective; QoL, quality of life; R, retrospective; TGD, transgender and gender-diverse.Arrow down: reduced; Arrow up: increased.

Data synthesis was then reported in the Result section in the form of a narrative summary, as meta-analysis was not possible. Evidence statements were drawn whether evidence were available.

All data included in this review derive from published peer-reviewed studies. No unpublished or proprietary data were used. The authors confirm that all data are appropriately cited.

### Terminology

The classification of GD is considered by some to be stigmatizing due to its pathologizing nature, whereas GI is viewed as a less medicalized term. However, the majority of published studies still refer to GD, and in some countries, including Italy, access to treatment is only possible with a formal GD diagnosis. To maintain consistency and inclusivity, we have aimed to minimize the use of both terms and have instead prioritized the use of “TGD adolescents” throughout the manuscript wherever possible.

## Results

### Search results

A total of 815 studies were identified following the literature search after duplicates were removed. After reviewing titles and abstracts, 636 additional records were excluded: 234 review articles, 15 guidelines, 56 studies including only participants older than 18 years, 302 studies reporting outcomes different from those of interest, 7 studies not available as full papers, 21 studies with the number of TGD adolescents <10, 1 study with publication period before 2011.

**Figure 1:**
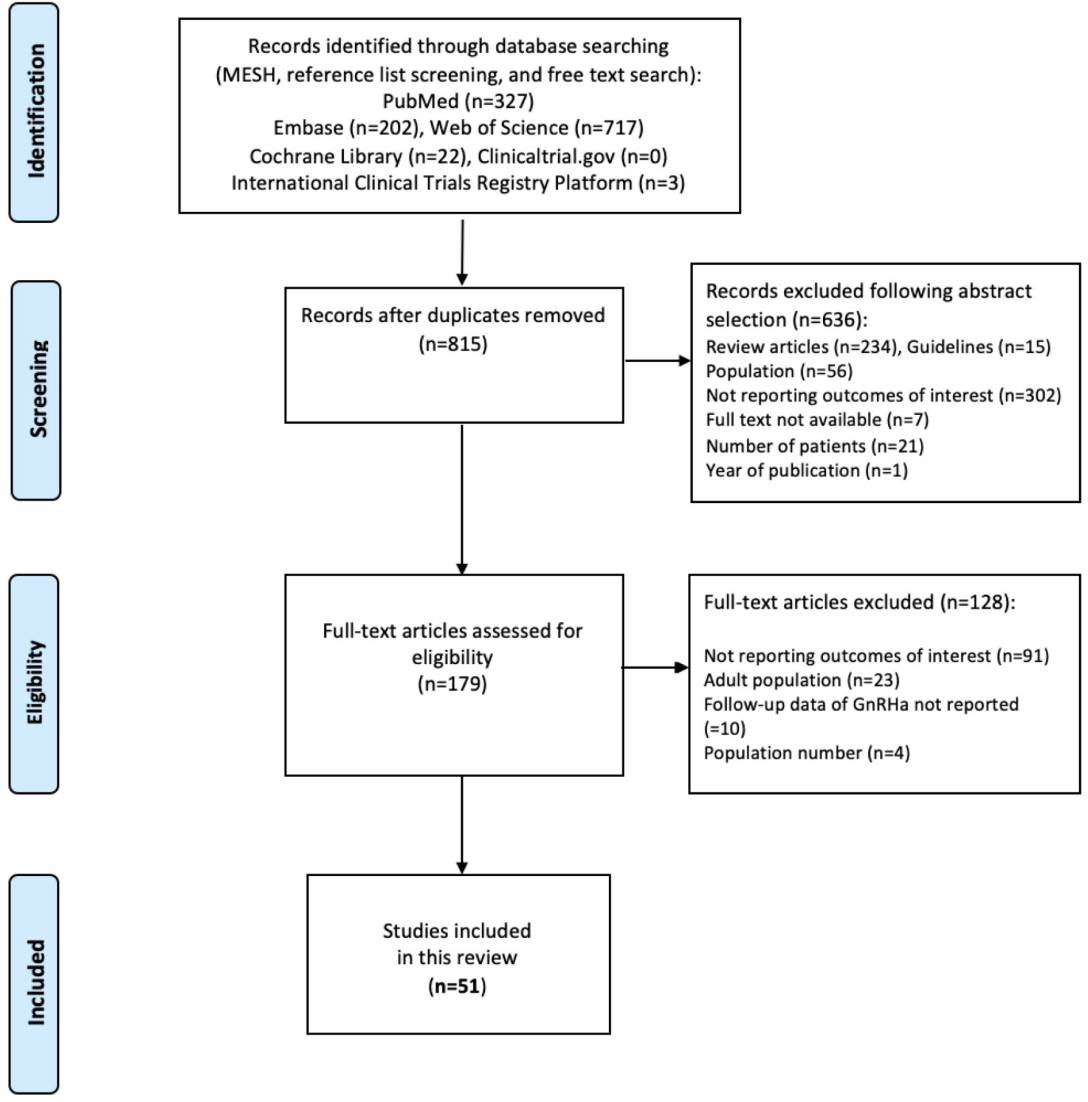
PRISMA flow diagram.

A total of 179 full-text manuscripts were assessed for eligibility. After full-text examination, 128 studies were excluded: 91 not reporting outcomes of interest (most not including data on GnRHa), 23 regarding only the adult population, 10 not presenting follow-up data of GnRHa treatment, and 4 reporting <10 TGD adolescents treated with GnRHa. The PRISMA flow diagram (**Figure 1**) summarizes the publication screening process. The list of excluded papers and the reasons for exclusion are reported in **Supplementary Material S2**.

Fifty-one studies (4, 10–59) were finally included in this systematic review. The summary and grade of the evidence of each study are reported in **Supplementary Tables 1A–D**. Among the selected studies, 17 analyzed physical and hormone levels (**Supplementary Table 1A**), 10 bone outcomes (**Supplementary Table 1B**), 20 mental health outcomes (**Supplementary Table 1C**), 3 about fertility and sexual function outcomes, and 7 focused on side effects and risk of cancer (**Supplementary Table 1D**). All the studies included were in English. Because of the heterogeneity of study populations and outcomes, results could not be combined in a meta-analysis.

Among the selected studies, none were RCTs, 19 were prospective longitudinal, 27 were retrospective, and 5 were cross-sectional in design. The number of TGD adolescents treated with GnRH in the studies varied between 13 and 410 (33, 34). The duration of follow-up in GnRHa-treated individuals ranged between 6 months to 22 years (31, 35).

There has been a steady increase in publications over the years: 1 article in 2011; 2 in 2014, 2 in 2015, 1 in 2016, 1 in 2017, 3 in 2018, 1 in 2019, 12 in 2020, 7 in 2021, 6 in 2022, 12 in 2023 and 3 in 2024 until February 1, 2024.

The level of certainty was decreased by one or two levels for these reasons:

- sample size (median values as cut-off): studies including less than 121 individuals treated with GnRHa for the physical-hormonal outcomes, 50 for the mental health outcomes, 72.5 for the bone outcome, 78 for the fertility and sexual function outcome, 214 for side effects and risk of cancer outcome;
- possible bias due to the study design: cross-sectional or retrospective, p-values not shown, short follow-up (median values as cut-off: 3 years for the physical-hormonal outcomes, 1.5 years for the mental health outcomes, 2 years for the bone outcome, 1.45 years for side effects and risk of cancer outcome), no control group;
- imprecision of estimates or inconsistent results across different cohorts included in multicenter studies.

**Tables 3A**-**C** summarize the 22 studies with moderate-high quality levels. Below, we report the main results according to moderate-high-level divided by outcome (4, 10–30). No comments on fertility, sexual function, side effects, or cancer risk will be provided, as none of the studies met a moderate or high level of evidence. We explored heterogeneity by conducting subgroup analyses, based on population characteristics (age, Tanner stage), modality of treatment (GnRHa, GAHT), and treatment duration (3 months to 3 years). This analysis led to results for the outcomes height, Body Mass Index (BMI), BMD and Global Function, Cognition, and Behavioral/Emotional Problems, while for others only a few studies were available for each subgroup. **Table 4** further summarizes the main findings of the review.

**Table 4:** Summary of findings.

Outcome Category	Key Findings	Study Limitations	Clinical Implications
Physical Changes & Metabolic Outcomes	GnRHa effectively suppresses puberty. Height velocity decreases during treatment, but final height aligns with target height. Changes in body composition observed: increase in fat mass, reduction in lean body mass, and a transient BMI increase in AFAB individuals. Some adolescents report dissatisfaction with final height when it does not align with the average for their affirmed gender.	Variability in treatment protocols, GnRHa monotherapy duration, and age at initiation complicates predictions on final height and body composition.	Regular monitoring of growth and body composition is necessary. Patients should be counseled about possible height outcomes. Nutritional and physical activity recommendations should be integrated to manage changes in fat and lean body mass.
Bone Health	BMD decreases during GnRHa treatment, particularly in AMAB individuals. Recovery during GAHT is partial, and concerns remain about long-term skeletal health. No increased osteoporosis incidence has been observed. Bone turnover markers indicate suppressed bone activity.	Lack of standardized long-term follow-up studies limits understanding of adulthood skeletal outcomes.	Bone health monitoring should be a priority. Clinicians should encourage lifestyle measures to support bone health, including weight-bearing exercise and vitamin D supplementation. Long-term follow-up is needed to assess osteoporosis risk.
Mental Health	Significant improvements in global functioning, reduction in emotional and behavioral problems, and decrease in suicidality among adolescents receiving GnRHa, especially when followed by GAHT. Depression and anxiety levels drop significantly post-treatment, but body dissatisfaction and anxiety persist in some cases, particularly among AFAB individuals.	Limited data on long-term psychiatric outcomes.	Mental health support should be integrated throughout the transition process. While GnRHa shows clear benefits for suicidality and depression, continued psychological care is essential to address body image concerns and residual anxiety.

AFAB, assigned female at birth; AMAB, assigned male at birth; BMI, body mass index; MBD, bone mineral density; GAHT, gender-affirming hormone therapy; GnRHa, gonadotrophin releasing-hormone agonist; TGD, transgender and gender-diverse.

**Table 5:** Clinical monitoring recommendations for TGD adolescents undergoing GnRHa therapy.

1. Physical changes and hormone levels
• **Anthropometric Measures**: Monitor height, weight, and body mass index (BMI) every 6–12 months, with attention to changes in growth velocity and deviations from expected growth curves based on mid-parental height. • **Pubertal Status**: Document Tanner stage at baseline and follow-up, assessing suppression of secondary sex characteristics. • **Laboratory Evaluation**: Regular measurement (every 6–12 months) of gonadotropins (LH/FSH), sex steroids (estradiol or testosterone), and adrenal androgens (e.g., DHEAS, androstenedione) to confirm adequate suppression. • **Body Composition**: Consider periodic assessment (e.g., annually) of lean/fat mass using tools such as bioimpedance or DEXA, especially if BMI changes significantly. • **Transition to GAHT**: Assess readiness and timing for initiating gender-affirming hormone therapy, ensuring appropriate informed consent and psychosocial support.
2. Bone health
• **BMD Monitoring**: Perform DEXA scans every 12–24 months, focusing on lumbar spine and femoral neck Z-scores. • **Bone Turnover Markers**: Optional use of markers such as P1NP and CTX/1CTP to evaluate bone remodeling activity during suppression. • **Risk Mitigation**: Encourage regular weight-bearing physical activity and ensure adequate intake of calcium and vitamin D. Supplementation should be prescribed when needed. • **Watchpoints**: Monitor for additional risk factors such as low BMI, nutritional deficits, family history of osteoporosis, or long periods of suppression before GAHT initiation.
3. Mental health and psychosocial support
• **Baseline Assessment**: Conduct a comprehensive psychological evaluation before treatment initiation to identify pre-existing mental health conditions. • **Ongoing Support**: Provide continuous access to affirming mental health services throughout GnRHa treatment and beyond, including individual or family therapy. • **Monitoring Tools**: Use standardized tools (e.g., CBCL, YSR) to track emotional and behavioral outcomes over time. • **Watchpoints**: Pay special attention to persistent body dissatisfaction, anxiety, or depressive symptoms, particularly in AFAB youth.
4. Fertility and sexual function
• **Fertility Counseling**: Discuss potential impact of GnRHa and future GAHT on fertility before starting treatment, even if adolescents are young or undecided. • **Preservation Options**: Refer to fertility specialists early for options such as sperm or oocyte cryopreservation (depending on Tanner stage and consent). • **Follow-Up**: Continue to revisit the topic as adolescents age or approach transition to GAHT or adulthood.
5. Cancer risk and other adverse effects
• **Cancer Surveillance**: While direct evidence is lacking, encourage regular clinical follow-up with physical exams and targeted evaluations as clinically indicated. • **Long-Term Monitoring**: Document any adverse effects, including rare events (e.g., idiopathic intracranial hypertension), and collect data in registries or longitudinal cohorts to support future evidence generation. • **Breast/Prostate Considerations**: In post-GAHT individuals, tailor cancer screening recommendations according to affirmed gender and anatomy.
6. Cognitive and educational monitoring (optional but recommended)
• **Cognitive Development**: For adolescents with neurodevelopmental conditions or learning differences, consider baseline and follow-up neuropsychological evaluations. • **Educational Support**: Collaborate with schools to provide appropriate accommodations if cognitive or psychosocial challenges are identified during treatment.
7. Ethical and informed consent
• **Shared Decision-Making**: Ensure informed consent processes are developmentally appropriate, inclusive of both adolescent and caregivers, and revisited regularly. • **Documentation**: Maintain thorough records of discussions around benefits, risks, unknowns, and the adolescent’s evolving goals.

Given the current evidence, we recommend a multidisciplinary and personalized approach to the clinical monitoring of adolescents undergoing GnRHa therapy. The following areas should be regularly evaluated: AFAB, Assigned Female at Birth; BMD, Bone Mineral Density; BMI, Body Mass Index; CBCL, Child Behavior Checklist; CTX/1CTP, Cross-linked Telopeptide of Type 1 Collagen; DEXA, Dual-Energy X-ray Absorptiometry; DHEAS, Dehydroepiandrosterone Sulfate; FSH, Follicle-Stimulating Hormone; GAHT, Gender-Affirming Hormone Therapy; GnRHa, Gonadotropin-Releasing Hormone agonists; LH, Luteinizing Hormone; P1NP, Procollagen Type 1 N-Terminal Propeptide; TGD, Transgender and Gender Diverse; YSR, Youth Self Report.

### Physical changes and hormone levels

- Height: during GnRHa treatment, significant decreases in height Standard Deviation Score (SDS) were observed in both AMAB and AFAB individuals, particularly in the first 1 to 2 years (14). In one study, only AMAB adolescents showed a progressive reduction in height percentiles (4). Height velocity (HV) also decelerated during treatment (10, 16), with no significant difference between AMAB and AFAB individuals (16). However, the Tanner stage at the start of treatment played a crucial role, as starting GnRHa later was associated with significantly lower HV (16). Final height (FH) outcomes were mixed. Van de Grift et al. found no statistical difference in FH among those treated early, later, or not with GnRHa (18). Boogers et al. reported that in AMAB adolescent FH was slightly lower than predicted at the start of GnRHa but not significantly different from the target height (TH) (10). High doses of estradiol, particularly ethinylestradiol, further reduced FH below predicted values and TH. However, Willemsen et al. noted that, after a period of catch-up growth during GAHT, FH in AFAB individuals exceeded mid-parental height (MPH) by an average of 3.9± 6 cm (19). Thus, the start of GnRHa in early-mid puberty followed by GAHT alters growth velocity in TGD youth, and the individual height gain after the start of GAHT is difficult to predict. However, as shown by Ciancia et al. the medical trajectory does not seem to alter FH in both AMAB and AFAB, for whom FH is in line with TH calculated for the sex at birth (32).
- Sexual characteristics and gonadotropins/sexual steroids: GnRHa reduces the development of sex characteristics (breast or testicular volume) in TGD adolescents (4, 14, 18), and this data is confirmed by gonadotropin and sex steroid hormone suppression (4, 14). As a result, AFAB may not need to undergo mastectomy, while AMAB may require an alternative to penile inversion vaginoplasty (18). Dehydroepiandrosterone sulfate (DHEAS) levels remained stable during treatment, while androstenedione decreased during GnRHa but rose during GAHT in AFAB, with no changes in AMAB (15).
- Body composition and serum hormone profile: during GnRHa treatment, increases in total body fat and gynoid fat and reduction in lean mass without significant changes in BMI SDS were observed in AMAB adolescents, whereas in AFAB adolescents, no significant changes in BMI, lean body mass or total body fat Z-score were observed (13). In another study, a significant but transient increase in BMI percentiles was observed in AFAB individuals, beginning in the third month of therapy and lasting only a few months (4). When considering subsequent GAHT, body composition shifted toward the affirmed sex (11), with a decrease in lean body mass and an increase in fat mass (14). Obesity prevalence was higher in TGD individuals compared to cisgender controls, but lipid profiles and insulin sensitivity remained comparable to the general population (12). TGD adolescents had higher odds of overweight/obesity; however, GnRHa alone and estradiol were not associated with greater odds of cardiometabolic-related diagnoses, while those treated with testosterone, had a higher risk of dyslipidemia and liver dysfunction (17).

### Bone health

- Bone mineral density: in a few studies BMD Z-scores at lumbar spine (LS) and femoral neck (FN) decreased during GnRHa treatment and increased during GAHT (22, 23). This finding was not confirmed by another study which did not find any changes in BMD Z-scores at the LS and FN during GnRHa (20), probably due to the relatively short follow-up duration. Of note, AMAB often presents with low BMD z-scores already before the start of any gender-affirming treatment and the low BMD Z-scores (mainly at LS) can persist also after 3 years of GAHT (21).
- Bone turnover markers: the added value of evaluating bone turnover markers seems to be limited. However, decreased levels of Procollagen type 1 N-terminal propeptide (P1NP), as a marker of bone formation, and of cross-linked telopeptide of type 1 collagen (1CTP), as a marker of bone resorption, indicate decreased bone turnover during GnRHa treatment (21, 23).

### Mental health

- Global function, cognition, and behavioral/emotional problems: adolescents undergoing GnRHa treatment, especially when followed by GAHT, showed improvements in global functioning and reductions in emotional and behavioral problems over time; these results are more evident for AMAB adolescents (4, 27). For instance, improvements in psychosocial functioning were noted, with those receiving both psychological support and GnRHa showing better and improved outcomes steadily over time, than those receiving psychological support alone (26). Cognitive outcomes, including IQ, were not significantly affected by gender-affirming treatment, and post-treatment educational achievement was largely linked to pre-treatment cognitive scores (25). While behavioral and emotional issues persisted in some cases, the overall trajectory indicated improvement, particularly in global functioning scores (27, 28).
- Suicide ideation: GnRHa treatment has been shown to significantly reduce suicidality in transgender adolescents. In a study comparing transgender adolescents at referral and those using GnRHa, the endorsement of self-harm/suicidality in those who started GnRHa (12.4%) was lower than in those at referral (27.2%) and aligned closely with the rate in cisgender peers (11.9%) (30). Adolescents who received either GnRHa or GAHT experienced a 73% lower risk of suicidality compared to those who did not undergo hormone treatment (29). The reduction in suicidal risk was positively associated with the physical effects and the hormone changes (4). Additionally, Achille et al. reported that suicidal ideation decreased significantly over time across all groups receiving GnRHa or GAHT compared to baseline (from 10% to 6%) (24).
- Depression/anxiety: while at referral, 31.3% of transgender adolescents exhibited clinical levels of internalizing problems, including depression and anxiety, with higher rates in AMAB (35.3%) compared to AFAB (28.2%), after initiating GnRHa these rates dropped to 16.3%, bringing them closer to those of cisgender peers (30). Across multiple studies, both depression and anxiety levels decreased significantly over time with affirmative treatment, with transgender youth showing marked improvements (24, 27, 28); adolescents receiving GnRHa or GAHT had a 60% lower risk of depression compared to those who did not undergo treatment (29). Unlike psychological assessment alone, GnRHa therapy correlated strongly with decreases in anxiety, alongside observed hormonal and physical changes (4).
- Quality of life/well-being: Quality of life (QoL) improved significantly in adolescents who underwent GnRHa treatment, particularly when followed by GAHT and surgery. Emotional, social, and physical well-being scores improved over time, aligning with those of cisgender controls post-treatment (28). Adolescents reported better overall life satisfaction and well-being, though some studies noted that improvements were gradual, particularly in the early stages of treatment (24). While some studies demonstrated gradual improvements in well-being, particularly following surgery, others showed substantial enhancement in overall life satisfaction (26, 28).
- Body dissatisfaction: Gender dysphoria and body image satisfaction remained unchanged during GnRHa, with AFAB individuals reporting higher levels of dissatisfaction with their primary and secondary sex characteristics, compared to AMAB individuals (27). Body dissatisfaction persisted during GnRHa treatment but significantly improved after GAHT and surgery (28). Recently, Fisher et al. reported that adolescents were less worried about body changes once they started GnRHa, and the reduction in body uneasiness was more evident in AMAB adolescents (4).

## Discussion

The findings of this systematic review contribute to the growing body of literature on the efficacy and safety of GnRHa for puberty suppression in TGD adolescents with or without GAHT. The review synthesizes data from 51 studies, highlighting both the physical, psychological, and metabolic outcomes of GnRHa in TGD adolescents, incorporating evidence from 22 studies with moderate to high-quality levels.

While previous systematic reviews have identified gaps in the literature, including limited evidence on long-term psychosocial outcomes and inconsistencies in study designs (6, 7), this review adds clarity by focusing on more recent studies and categorizing the outcomes based on their level of evidence, as well as exploring their clinical significance (**Table 4**). Furthermore, the increasing number of publications in recent years highlights a growing body of research, aiming at high-quality and evidence-based medicine also in this specific field.

However, the impact of GnRHa treatment on physical development, particularly height, body composition, and bone health, remains not completely understood, although increasing evidence supports the safety of this treatment as part of gender-affirming care. As mentioned by previous studies (6), GnRHa effectively suppresses the further development of secondary sexual characteristics, contributing to the reduction of dysphoria. Nevertheless, this treatment alters the linear growth pattern, especially when started during early or mid-puberty. However, the combination with subsequent GAHT compensates for the initial decrease in height velocity (10, 14, 16) and TGD youth tend to reach a FH in line with their TH calculated for the sex at birth (32). From a clinical perspective, these findings suggest that while GnRHa supports gender-affirming care, it is crucial to set realistic expectations regarding final height, particularly for adolescents who desire an FH more aligned with the average for their experienced gender.

Body composition changes observed during GnRHa treatment, such as increases in fat mass and reductions in lean body mass, were consistent among studies (11, 14). While body composition shifts towards the affirmed sex during GAHT, there is a higher prevalence of obesity among TGD adolescents compared to cisgender controls (12). Interestingly, lipid profiles and insulin sensitivity remained comparable to the general population, suggesting that the cardiometabolic risks may be more closely associated with testosterone use rather than GnRHa alone (17). This distinction was not highlighted in earlier reviews and suggests a need for more tailored metabolic monitoring in TGD individuals, particularly those on testosterone therapy.

Bone health remains an area of concern, as GnRHa treatment is associated with reductions in BMD (21, 22). These findings are consistent with earlier reviews (6), but the evidence provided here indicates that BMD improves during GAHT, albeit not always to pre-treatment levels, especially in AMAB individuals (notably, AMAB adolescents often present with low BMD Z-scores even before initiating any gender-affirming treatment, indicating a possible baseline vulnerability that may influence long-term bone outcomes) (21). This raises concerns about the potential long-term impact on skeletal health, especially in those who undergo prolonged periods of puberty suppression. However, so far there is no evidence of increased osteoporosis incidence during childhood, defined as BMD below normal values for age and gender (Z-score ≤ −2) accompanied by a history of repeated fractures (60). If the treatment would increase the risk of osteoporosis later in life is also still to be understood. The determination of bone turnover markers (P1NP, 1CTP) has been proposed to better characterize bone remodeling during GnRHa treatment, but it has shown limited utility, although a decrease in these markers has been registered, indicating suppressed bone activity during the treatment (21, 23). This findings reinforce the need for a multifaceted approach to monitor bone health, especially in cases with multiple risk factors, but above all to increase measures aimed at promoting the maintenance of good bone health (e.g. physical activity, balanced diet, vitamin D supplementation).

Previous reviews reported that the impact of GnRHa on mental health remains a central focus of debate (6–8), as a few studies of low or moderate quality of evidence regarding psychological and cognitive outcomes were included (8). Compared to earlier reviews, this study incorporates more recent data, demonstrating significant improvements in global functioning, depression, and anxiety, with studies reporting a reduction in suicidal ideation and self-harm in TGD adolescents also receiving GAHT (24, 29). These improvements are critical, given that TGD adolescents often experience elevated rates of mental health issues prior to treatment (61–63). The substantial decrease in self-harm ideation aligns with reports that suggest affirmative treatments can mitigate mental health challenges faced by transgender youth (4, 30). Importantly, the reduction in suicidality observed in adolescents using GnRHa alone compared to those at referral (30) and also to psychological assessment alone (4) provides stronger evidence supporting the protective role of puberty suppression in transgender youth, a point that had been largely hypothesized but not robustly evidenced in earlier reviews.

While the reduction in depressive symptoms was consistently reported across multiple studies (24, 28), this review also highlights that anxiety and body dissatisfaction persist during GnRHa treatment (4), particularly in AFAB individuals (27). Additionally, the association between improved mental health outcomes and the initiation of GAHT or surgery suggests that while GnRHa is effective in halting the distressing progression of puberty, the long-term benefits on mental health may be realized more fully with comprehensive gender-affirming treatment.

Furthermore, our results show that QoL improves significantly following GnRHa treatment, particularly when followed by GAHT. This finding supports earlier studies indicating enhanced psychosocial functioning among adolescents who receive comprehensive gender-affirming care (28).

The strengths of this review include its focus on moderate to high-quality evidence, providing a clearer understanding of the outcomes associated with GnRHa treatment in TGD adolescents. By excluding studies with low levels of evidence, this review presents more reliable conclusions than earlier reviews that included studies with significant methodological limitations. Overall, this study builds upon previous systematic reviews by providing a more comprehensive analysis of the current evidence on GnRHa treatment for TGD adolescents, highlighting both its benefits and potential risks. Unlike earlier reviews (6, 7), which primarily focused on physical outcomes with limited discussion of psychosocial aspects, this study offers a broader examination of both efficacy and adverse effects, including physical changes, mental health, bone health, and psychosocial outcomes. Furthermore, this study employs a rigorous GRADE assessment to evaluate the quality of evidence for each outcome across included studies, enhancing its reliability compared to previous reviews. While earlier reviews pointed to insufficient data on mental health impacts, this study emphasizes significant improvements in mental health outcomes, quality of life, and reductions in suicidality among adolescents receiving GnRHa treatment. Additionally, this study addresses concerns regarding bone health more thoroughly than prior reviews, specifically highlighting findings related to decreased BMD during treatment. Lastly, this review includes a substantial amount of longitudinal data, which was either absent in Chew et al. or only partially covered in Ludvigsson’s analysis (6, 7).

However, the review has some limitations. One key limitation is that a meta-analysis was not conducted. This decision was made due to the significant heterogeneity in study designs, treatment protocols, follow-up durations, and outcome measures across the included studies. The variation in how outcomes were assessed and reported made it challenging to synthesize the data into a meaningful pooled estimate. Additionally, differences in the populations studied, including variations in age at treatment initiation and duration of GnRHa use, further limited the feasibility of conducting a meta-analysis. This heterogeneity may impact the generalizability of findings and clinicians must be aware of the diverse results and factors that could influence treatment decisions. Moreover, the lack of RCTs and the reliance on observational and retrospective studies introduce potential biases that could affect the robustness of the conclusions. Additionally, publication bias may be a factor, as studies reporting positive outcomes may be more likely to be published, potentially skewing the overall interpretation of results.

Given the existence of 22 studies with moderate to high levels of evidence so far, it seems inaccurate to assert that there is still insufficient and/or inconsistent evidence about the effects of puberty suppression in TGD adolescents (8) or that GnRH treatment should only be offered under a research protocol due to a lack of solid evidence on long-term outcomes for managing gender-related distress (64). GnRHa has been safely and effectively used to treat central precocious puberty (CPP) since 1981, despite their approval being based on relatively short, open-label studies involving small patient populations. A recent systematic review (65) found only 98 studies (with no RCTs), 98.5% of which involved girls (meaning that there is very limited literature supporting male cases): 18 were comparative studies (with only 13 comparing against no treatment), while the remaining 81 were single-arm studies. Conducting more rigorous studies, such as RCTs, would be considered at this point neither feasible nor ethical in CPP as in TGD adolescents (66). While medicine constantly strives for more robust evidence, it would be unethical to deny or discontinue the use of GnRHa in TGD adolescents when substantial evidence demonstrates its benefits.

Clinical decisions, particularly in pediatrics, where the diseases can be rare and drugs can be not yet approved, are rarely based solely on RCTs. A recent study found that over 90% of healthcare interventions reviewed in Cochrane Reports lack high-quality evidence (67). This highlights the broader challenge in medicine where decisions often must be made in the face of imperfect evidence, relying on a combination of clinical experience, patient preferences, and available data. In cases where strong evidence is still lacking, it becomes essential to outline the appropriate approach for therapeutic choices and monitoring, based on regularly updated systematic reviews that weigh the significance of available publications.

The use of hormone therapy in TGD adolescents continues to be a topic of discussion; however, prioritizing the mental and physical health of TGD youth, who are more vulnerable to psychological challenges, remains crucial. Healthcare professionals should base treatment decisions on the specific needs and context of each individual, with a foundation in scientific research rather than personal beliefs or societal biases. As in the Italian experience, GnRHa therapy is administered only in carefully selected cases at specialized centers, following thorough psychological assessment and reserved for those experiencing significant and enduring distress (68).

### Clinical implications and ethical considerations

Considering the increased importance of GnRHa treatment in this area, it is crucial to highlight several key clinical and ethical considerations related to the use of GnRHa in transgender and gender-diverse adolescents.

Given the observed decrease in bone mineral density during GnRHa treatment, especially in AMAB individuals, long-term monitoring of skeletal health is essential. Although no cases of osteoporosis have been reported during adolescence, the potential long-term risks remain uncertain, requiring continued vigilance.

While improvements in mental health are well documented, persistent anxiety and body dissatisfaction—particularly in AFAB individuals—highlight the need for ongoing psychological support throughout GnRHa treatment, a recommendation emphasized in earlier reviews but not always reflected in clinical practice.

Moreover, the long-term effects of GnRHa on fertility preservation, sexual function, and cancer risk remain understudied, as no studies with moderate or high evidence addressed these outcomes in this review. These remain important research priorities to inform future clinical guidelines.

Due to the sensitive nature of GnRHa treatment, a rigorous informed consent process involving both adolescents and their families is essential. This should include a thorough explanation of known or potential benefits, risks, uncertainties, and long-term consequences, allowing for shared decision-making (68, 69).

Finally, a precision medicine approach is crucial in managing GnRHa treatment, taking into account factors such as pubertal stage, psychological health, individual goals, and social context. Personalized care plans optimize outcomes while minimizing potential risks. In this context, we propose a structured framework for clinical monitoring to guide individualized care and ensure comprehensive, evidence-informed follow-up throughout the course of treatment (**Table 5**).

## Conclusions

This systematic review provides a comprehensive overview of current evidence on the use of GnRHa in TGD adolescents, confirming its efficacy in suppressing puberty and improving mental health outcomes. While long-term data remain limited, especially regarding bone health, fertility, and cancer risk, available moderate to high-quality evidence supports its clinical use as part of gender-affirming care.

In addition to clinical outcomes, several key considerations must guide practice. Bone health monitoring is essential, particularly in AMAB individuals who are at greater risk of reduced BMD during treatment. Ongoing psychological support should be provided throughout GnRHa use, especially for AFAB adolescents, given the persistence of body dissatisfaction and anxiety in some cases. Fertility preservation should be discussed before treatment initiation, despite the current lack of robust evidence.

A rigorous shared decision-making process involving adolescents and their families is critical, ensuring informed consent and personalized care based on individual needs and goals.

Future research should prioritize co-produced, longitudinal studies that address these gaps and further support ethical, safe, and evidence-based use of GnRHa in this population.

## Data Availability

The original contributions presented in the study are included in the article/**Supplementary Material**, Further inquiries can be directed to the corresponding author/s.
