# Supplementary material for: Use of gonadotropin-releasing hormone agonists in transgender and gender diverse youth: a systematic review
Source: Front Endocrinol (Lausanne). 2025 May 14;16:1555186. doi: 10.3389/fendo.2025.1555186 (PMC12116301; doi:10.3389/fendo.2025.1555186)
Supplement: Supplementary file 1 [file DataSheet1.docx]

**Supplementary material S1:** Exact terms used to search each database

**Pubmed, EMBASE, The Cochrane Library** (advanced search)
Limits: 0-18 years, Publication date 2011.

“Gender Dysphor*” or “Gender incongruen*” or transgender or nonbinary
AND “Gonadotropin-releasing hormone agonist” or “GnRH Analogue”

“Gender Dysphor*” or “Gender incongruen*” or transgender or nonbinary
AND “puberty block*” or “puberty suppress*” or “puberty inhibit*”

**Web of Science** (advanced search)

“Gender Dysphor*” or “Gender incongruen*” or transgender or nonbinary
AND “Gonadotropin-releasing hormone agonist” or “GnRH Analogue”

“Gender Dysphor*” or “Gender incongruen*” or transgender or nonbinary
AND “puberty block*” or “puberty suppress*” or “puberty inhibit*”

**Clinicaltrial.gov**

*Condition/Disease:* “Gender Dysphoria”
*Other terms:* “Gender Dysphor, Adolescents” or “Gender incongruence” or “trangdender individuals”

*Intervention/treatment:* “Gonadotropin-releasing hormone agonist\GNRH-A” or “GnRH Analogue” or “puberty block*” or “puberty suppress*” or “puberty inhibit*”

**International Clinical Trials Registry Platform (ICTRP)** (advanced search)
Recruitment status: ALL
With results only

“Gender Dysphor*” or “Gender incongruen*” or transgender or nonbinary
AND GnRH or “puberty block*” or “puberty suppress*” or “puberty inhibit*”
