# Supplementary material for: Use of gonadotropin-releasing hormone agonists in transgender and gender diverse youth: a systematic review
Source: Front Endocrinol (Lausanne). 2025 May 14;16:1555186. doi: 10.3389/fendo.2025.1555186 (PMC12116301; doi:10.3389/fendo.2025.1555186)
Supplement: Supplementary file 2 [file DataSheet2.doc]

**Supplementary Material S2: List of studies excluded at full-text screening stage, with brief reasons (No. 128)**

| **No** | **Title** | **Authors** | **Citation** | **Reason of exclusion** |
| --- | --- | --- | --- | --- |
| 1 | The Amsterdam Cohort of Gender Dysphoria Study (1972-2015): Trends in Prevalence, Treatment, and Regrets | Wiepjes CM, Nota NM, de Blok CJM, Klaver M, de Vries ALC, Wensing-Kruger SA, de Jongh RT, Bouman MB, Steensma TD, Cohen-Kettenis P, Gooren LJG, Kreukels BPC, den Heijer M. | J Sex Med. 2018 Apr;15(4):582-590. doi: 10.1016/j.jsxm.2018.01.016. | outcome |
| 2 | Blood Pressure Dynamics After Pubertal Suppression with Gonadotropin-Releasing Hormone Analogs Followed by Testosterone Treatment in Transgender Male Adolescents: A Pilot Study | Perl L, Segev-Becker A, Israeli G, Elkon-Tamir E, Oren A. | LGBT Health. 2020 Aug/Sep;7(6):340-344. doi: 10.1089/lgbt.2020.0026. | outcome |
| 3 | Breakthrough Bleeding in Transgender and Gender Diverse Adolescents and Young Adults on Long-Term Testosterone | Grimstad F, Kremen J, Shim J, Charlton BM, Boskey ER. | J Pediatr Adolesc Gynecol. 2021 Oct;34(5):706-716. doi: 10.1016/j.jpag.2021.04.004. | outcome |
| 4 | Mortality trends over five decades in adult transgender people receiving hormone treatment: a report from the Amsterdam cohort of gender dysphoria | De Blok CJ, Wiepjes CM, van Velzen DM, Staphorsius AS, Nota NM, Gooren LJ, Kreukels BP, den Heijer M. | Lancet Diabetes Endocrinol. 2021 Oct;9(10):663-670. doi: 10.1016/S2213-8587(21)00185-6. | outcome |
| 5 | Postoperative Vaginal Bleeding Concerns after Gender-Affirming Hysterectomy in Transgender Adolescents and Young Adults on Testosterone | Cipres DT, Shim JY, Grimstad FW. | J Pediatr Adolesc Gynecol. 2023 Feb;36(1):33-38. doi: 10.1016/j.jpag.2022.09.002. | outcome |
| 6 | Children and adolescents in the Amsterdam Cohort of Gender Dysphoria: trends in diagnostic- and treatment trajectories during the first 20 years of the Dutch Protocol | Van der Loos MATC, Klink DT, Hannema SE, Bruinsma S, Steensma TD, Kreukels BPC, Cohen-Kettenis PT, de Vries ALC, den Heijer M, Wiepjes CM. | J Sex Med. 2023 Feb 27;20(3):398-409. doi: 10.1093/jsxmed/qdac029. | outcome |
| 7 | Effects of three different testosterone formulations in female-to-male transsexual persons | Pelusi C, Costantino A, Martelli V, Lambertini M, Bazzocchi A, Ponti F, Battista G, Venturoli S, Meriggiola MC. | J Sex Med. 2014 Dec;11(12):3002-11. doi: 10.1111/jsm.12698. | outcome |
| 8 | Cross-sex hormonal treatment and body uneasiness in individuals with gender dysphoria | Fisher AD, Castellini G, Bandini E, Casale H, Fanni E, Benni L, Ferruccio N, Meriggiola MC, Manieri C, Gualerzi A, Jannini E, Oppo A, Ricca V, Maggi M, Rellini AH. | J Sex Med. 2014 Mar;11(3):709-19. doi: 10.1111/jsm.12413. | outcome |
| 9 | Menstrual Suppression in Adolescent and Young Adult Transgender Males | Alaniz VI, Sheeder JL, Whitmore GT, Wilde MD, Hutchens KJ, Nokoff NJ, Reirden DH, Huguelet PS. | J Pediatr Adolesc Gynecol. 2023 Apr;36(2):116-121. doi: 10.1016/j.jpag.2022.10.007. | outcome |
| 10 | Explorative Prospective Evaluation of Short-Term Subjective Effects of Hormonal Treatment in Trans People-Results from the European Network for the Investigation of Gender Incongruence | van Dijk D, Dekker MJHJ, Conemans EB, Wiepjes CM, de Goeij EGM, Overbeek KA, Fisher AD, den Heijer M, T'Sjoen G. | J Sex Med. 2019 Aug;16(8):1297-1309. doi: 10.1016/j.jsxm.2019.05.009. | outcome |
| 11 | Changes in regional body fat, lean body mass and body shape in trans persons using cross‐sex hormonal therapy: results from a multicenter prospective study | Klaver M, de Blok CJM, Wiepjes CM, Nota NM, Dekker MJHJ, de Mutsert R, Schreiner T, Fisher AD, T'Sjoen G, den Heijer M. | Eur J Endocrinol. 2018 Feb;178(2):163-171. doi: 10.1530/EJE-17-0496 | outcome |
| 12 | A randomized double‐blind placebo‐controlled pilot trial on the effects of testosterone undecanoate plus dutasteride or placebo on muscle strength, body composition, and metabolic profile in transmen. | Gava G, Armillotta F, Pillastrini P, Giagio S, Alvisi S, Mancini I, Morselli PG, Seracchioli R, Meriggiola MC. | J Sex Med. 2021 Mar;18(3):646-655. doi: 10.1016/j.jsxm.2020.12.015. | outcome |
| 13 | Body composition, bone turnover, and bone mass in trans men during testosterone treatment: 1‐year follow‐up data from a prospective case‐controlled study (ENIGI). | Van Caenegem E, Wierckx K, Taes Y, Schreiner T, Vandewalle S, Toye K, Lapauw B, Kaufman JM, T'Sjoen G. | Eur J Endocrinol. 2015 Feb;172(2):163-71. doi: 10.1530/EJE-14-0586. | outcome |
| 14 | Efficacy and safety of pubertal induction using 17β-estradiol in transgirls. | Hannema SE, Schagen SEE, Cohen-Kettenis PT, Delemarre-Van De Waal HA | J Clin Endocrinol Metab. 2017 Jul 1;102(7):2356-2363. doi: 10.1210/jc.2017-00373. | outcome |
| 15 | Oxandrolone use in trans-masculine youth appears to increase adult height: preliminary evidence. | Grimstad FW, Knoll MM, Jacobson JD. | LGBT Health. 2021 May-Jun;8(4):300-306. doi: 10.1089/lgbt.2020.0355. | outcome |
| 16 | Incident diabetes risk is not increased in transgender individuals using hormone therapy. | van Velzen D, Wiepjes C, Nota N, van Raalte D, de Mutsert R, Simsek S, den Heijer M. | J Clin Endocrinol Metab. 2022 Apr 19;107(5):e2000-e2007. doi: 10.1210/clinem/dgab934. | outcome |
| 17 | Gender-affirming hormone therapy and risk of diabetes in transgender persons | Tangpricha V. | J Clin Endocrinol Metab 2022;107(6):e2632–e2633. doi: 10.1210/clinem/dgac060. | outcome |
| 18 | Concomitant psychiatric problems and hormonal treatment induced metabolic syndrome in gender dysphoria individuals: a 2 year follow-up study. | Colizzi M, Costa R, Scaramuzzi F, Palumbo C, Tyropani M, Pace V, Quagliarella L, Brescia F, Natilla LC, Loverro G, Todarello O | J Psychosom Res. 2015 Apr;78(4):399-406. doi: 10.1016/j.jpsychores.2015.02.001. | outcome |
| 19 | A long-term follow-up study of mortality in transsexuals receiving treatment with cross-sex hormones | Asscheman H, Giltay EJ, Megens JA, de Ronde WP, van Trotsenburg MA, Gooren LJ. | Eur J Endocrinol 2011;164(4): 635–42. doi: 10.1530/EJE-10-1038. | outcome |
| 20 | Subcutaneous Testosterone: An Effective Delivery Mechanism for Masculinizing Young Transgender Men. | Olson, J., Schrager, S. M., Clark, L. F., Dunlap, S. L., & Belzer, M. (2014 | *LGBT health*, *1*(3), 165–167. https://doi.org/10.1089/lgbt.2014.0018 | outcome |
| 21 | Consecutive cyproterone acetate and estradiol treatment in late-pubertal transgender female adolescents. | Tack LJW, Heyse R, Craen M, et al. | J Sex Med. 2017;14(5):747–757. doi: 10.1016/j.jsxm.2017.03.251. | outcome |
| 22 | Consecutive lynestrenol and cross- sex hormone treatment in biological female adolescents with gender dysphoria: a retrospective analysis. | Tack LJ, Craen M, Dhondt K, Vanden Bossche H, Laridaen J, Cools M. | Biol Sex Differ. 2016 Feb 16;7:14. doi: 10.1186/s13293-016-0067-9. | outcome |
| 23 | Breast Development in Transwomen After 1 Year of Cross-Sex Hormone Therapy: Results of a Prospective Multicenter Study. | De Blok CJM, Klaver M, Wiepjes CM, Nota NH, Heijboer AC, Fisher AD, et al. | J Clin Endocrinol Metab (2018) 103(2):532–8. doi: 10.1046/j.1365-2265.2003.01753.x | outcome |
| 24 | Sustained Breast Development and Breast Anthropometric Changes in 3 Years of Gender Affirming Hormone Treatment. | De Blok CJM, Dijkman B, Wiepjes C, Staphorius A, Timmermmans F, Smit J, et al. | J Clin Endocrinol Metab (2021) 106(2):e782–90. doi: 10.1210/jc.2017-01643 | outcome |
| 25 | Cross-Sex Hormone Therapy in Transgender Persons Affects Total Body Weight, Body Fat and Lean Body Mass: A Meta-Analysis. | Klaver M, Dekker MJHJ, de Mutser R, Twisk JWR, den Heijer M. | Andrologia (2017) 49(5):e12660. doi: 10.1210/jcem.83.2.4574 | outcome |
| 26 | Medroxyprogesterone Acetate in Gender- Affirming Therapy for Transwomen: Results From a Retrospective Study. | Jain J, Kwan D, Forcier M. | J Clin Endocrinol Metab (2019) 104(11):5148–56. doi: 10.1210/jc.2018-02253 | outcome |

| 27 | Binary and Non-binary Gender Identities, Internalizing Problems, and Treatment Wishes Among Adolescents Referred to a Gender Identity Clinic in Germany | Herrmann L, Barkmann C, Bindt C, Fahrenkrug S, Breu F, Grebe J, Becker-Hebly I. | Arch Sex Behav. 2024 Jan;53(1):91-106. doi: 10.1007/s10508-023-02674-8. | outcome |
| --- | --- | --- | --- | --- |
| 28 | Continuation of gender-affirming hormones in transgender people starting puberty suppression in adolescence: a cohort study in the Netherlands | van der Loos MATC, Hannema SE, Klink DT, den Heijer M, Wiepjes CM | Lancet Child Adolesc Health. 2022 Dec;6(12):869-875. doi: 10.1016/S2352-4642(22)00254-1. | outcome |
| 29 | Medical decision-making competence regarding puberty suppression: perceptions of transgender adolescents, their parents and clinicians | Vrouenraets LJJJ, de Vries ALC, Arnoldussen M, Hannema SE, Lindauer RJL, de Vries MC, Hein IM. | Eur Child Adolesc Psychiatry. 2023 Nov;32(11):2343-2361. doi: 10.1007/s00787-022-02076-6. | outcome |
| 30 | Assessing Medical Decision-Making Competence in Transgender Youth | Vrouenraets LJJJ, de Vries ALC, de Vries MC, van der Miesen AIR, Hein IM. | Pediatrics. 2021 Dec 1;148(6):e2020049643. doi: 10.1542/peds.2020-049643. | outcome |
| 31 | Transgender Dependent Adolescents in the U.S. Military Health Care System: Demographics, Treatments Sought, and Health Care Service Utilization | Van Donge N, Schvey NA, Roberts TA, Klein DA. | Mil Med. 2019 May 1;184(5-6):e447-e454. doi: 10.1093/milmed/usy264. | No follow up data |
| 32 | Perceptions of Sex, Gender, and Puberty Suppression: A Qualitative Analysis of Transgender Youth | Vrouenraets LJ, Fredriks AM, Hannema SE, Cohen-Kettenis PT, de Vries MC. | Arch Sex Behav. 2016 Oct;45(7):1697-703. doi: 10.1007/s10508-016-0764-9. | No follow up data |
| 33 | Youth and Caregiver Perspectives on Barriers to Gender-Affirming Health Care for Transgender Youth | Gridley SJ, Crouch JM, Evans Y, Eng W, Antoon E, Lyapustina M, Schimmel-Bristow A, Woodward J, Dundon K, Schaff R, McCarty C, Ahrens K, Breland DJ. | J Adolesc Health. 2016 Sep;59(3):254-261. doi: 10.1016/j.jadohealth.2016.03.017. | No follow up data |
| 34 | Psychiatric comorbidity in gender dysphoric adolescents | de Vries AL, Doreleijers TA, Steensma TD, Cohen-Kettenis PT. | J Child Psychol Psychiatry. 2011 Nov;52(11):1195-202. doi: 10.1111/j.1469-7610.2011.02426.x. | No follow up data |
| 35 | Patients' Priorities Regarding Female-to-Male Gender Affirmation Surgery of the Genitalia-A Pilot Study of 47 Patients in Sweden | Jacobsson J, Andréasson M, Kölby L, Elander A, Selvaggi G. | J Sex Med. 2017 Jun;14(6):857-864. doi: 10.1016/j.jsxm.2017.04.005. | surgical outcomes |
| 36 | Quality of life improvement after chest wall masculinization in female-to-male transgender patients: A prospective study using the BREAST-Q and Body Uneasiness Test | Agarwal CA, Scheefer MF, Wright LN, Walzer NK, Rivera A. | J Plast Reconstr Aesthet Surg. 2018 May;71(5):651-657. doi: 10.1016/j.bjps.2018.01.003. 31. | surgical outcomes |
| 37 | What is "Nonbinary" and What Do I Need to Know? A Primer for Surgeons Providing Chest Surgery for Transgender Patients | Esmonde N, Heston A, Jedrzejewski B, Ramly E, Annen A, Guerriero J, Hansen J, Berli J. | Aesthet Surg J. 2019 Apr 8;39(5):NP106-NP112. doi: 10.1093/asj/sjy166. | surgical outcomes |
| 38 | Baseline Mental Health and Psychosocial Functioning of Transgender Adolescents Seeking Gender-Affirming Hormone Therapy | Kuper LE, Mathews S, Lau M. | J Dev Behav Pediatr. 2019 Oct/Nov;40(8):589-596. doi: 10.1097/DBP.0000000000000697. | No follow up data |
| 39 | "I Couldn't See a Downside": Decision-Making About Gender-Affirming Hormone Therapy | Daley T, Grossoehme D, McGuire JK, Corathers S, Conard LA, Lipstein EA. | J Adolesc Health. 2019 Aug;65(2):274-279. doi: 10.1016/j.jadohealth.2019.02.018. Epub 2019 Jun 10. | outcome |
| 40 | Increasing normality-persisting barriers: Current socio-demographic characteristics of 350 individuals diagnosed with gender dysphoria | Meyer G, Mayer M, Mondorf A, Herrmann E, Bojunga J. | Clin Endocrinol (Oxf). 2020 Mar;92(3):241-246. doi: 10.1111/cen.14140. | outcome |
| 41 | Autism trait prevalence in treatment seeking adolescents and adults attending specialist gender services | Lehmann K, Rosato M, McKenna H, Leavey G. | Eur Psychiatry. 2020 Mar 2;63(1):e23. doi: 10.1192/j.eurpsy.2020.23. | outcome |
| 42 | Trajectories of Adolescents Treated with Gonadotropin-Releasing Hormone Analogues for Gender Dysphoria | Brik T, Vrouenraets LJJJ, de Vries MC, Hannema SE. | Arch Sex Behav. 2020 Oct;49(7):2611-2618. doi: 10.1007/s10508-020-01660-8. | outcome |
| 43 | Behavioral Health Concerns and Eligibility Factors Among Adolescents and Young Adults Seeking Gender-Affirming Masculinizing Top Surgery | Boskey ER, Jolly D, Tabaac AR, Ganor O. | LGBT Health. 2020 May/Jun;7(4):182-189. doi: 10.1089/lgbt.2019.0213. | outcome |
| 44 | Psychosocial Characteristics of Transgender Youth Seeking Gender-Affirming Medical Treatment: Baseline Findings From the Trans Youth Care Study | Chen D, Abrams M, Clark L, Ehrensaft D, Tishelman AC, Chan YM, Garofalo R, Olson-Kennedy J, Rosenthal SM, Hidalgo MA. | J Adolesc Health. 2021 Jun;68(6):1104-1111. doi: 10.1016/j.jadohealth.2020.07.033. | No follow up data |
| 45 | Mental Health and Timing of Gender-Affirming Care | Sorbara JC, Chiniara LN, Thompson S, Palmert MR. | Pediatrics. 2020 Oct;146(4):e20193600. doi: 10.1542/peds.2019-3600. Epub 2020 Sep 21. | No follow up data |
| 46 | Individual Treatment Progress Predicts Satisfaction With Transition-Related Care for Youth With Gender Dysphoria: A Prospective Clinical Cohort Study | Nieder TO, Mayer TK, Hinz S, Fahrenkrug S, Herrmann L, Becker-Hebly I. | J Sex Med. 2021 Mar;18(3):632-645. doi: 10.1016/j.jsxm.2020.12.010. | outcome |
| 47 | Mental Healthcare Utilization of Transgender Youth Before and After Affirming Treatment | Hisle-Gorman E, Schvey NA, Adirim TA, Rayne AK, Susi A, Roberts TA, Klein DA. | J Sex Med. 2021 Aug;18(8):1444-1454. doi: 10.1016/j.jsxm.2021.05.014. | outcome |
| 48 | Testosterone treatment, internalizing symptoms, and body image dissatisfaction in transgender boys | Grannis C, Leibowitz SF, Gahn S, Nahata L, Morningstar M, Mattson WI, Chen D, Strang JF, Nelson EE. | Psychoneuroendocrinology. 2021 Oct;132:105358. doi: 10.1016/j.psyneuen.2021.105358. | No follow up data |
| 49 | Self-injurious and suicidal behaviour in a transsexual adolescent and young adult population, treated at a specialised gender identity unit in Spain | Modrego Pardo I, Gómez Balaguer M, Hurtado Murillo F, Cuñat Navarro E, Solá Izquierdo E, Morillas Ariño C. | Endocrinol Diabetes Nutr (Engl Ed). 2021 May;68(5):338-345. doi: 10.1016/j.endien.2020.04.009. | No follow up data |
| 50 | It Might Take Time: A Study on the Evolution of Quality of Life in Individuals With Gender Incongruence During Gender-Affirming Care | Pavanello Decaro S, Van Gils S, Van Hoorde B, Baetens K, Heylens G, Elaut E. | J Sex Med. 2021 Dec;18(12):2045-2055. doi: 10.1016/j.jsxm.2021.09.008. | outcome |
| 51 | Mental Health Outcomes in Transgender and Nonbinary Youths Receiving Gender-Affirming Care | Tordoff DM, Wanta JW, Collin A, Stepney C, Inwards-Breland DJ, Ahrens K. | JAMA Netw Open. 2022 Feb 1;5(2):e220978. doi: 10.1001/jamanetworkopen.2022.0978. | outcome |
| 52 | Fertility Desire and Motivation Among Individuals with Gender Dysphoria: A Comparative Study | Durcan E, Turan S, Bircan BE, Yaylamaz S, Okur I, Demir AN, Sulu C, Kara Z, Sahin S, Taze SS, Mefkure Ozkaya H, Kadioglu P. | J Sex Marital Ther. 2022;48(8):789-803. doi: 10.1080/0092623X.2022.2053617.. | outcome |
| 53 | Self-Perception of Transgender Adolescents After Gender-Affirming Treatment: A Follow-Up Study into Young Adulthood | Arnoldussen M, van der Miesen AIR, Elzinga WS, Alberse AE, Popma A, Steensma TD, de Vries ALC. | LGBT Health. 2022 May-Jun;9(4):238-246. doi: 10.1089/lgbt.2020.0494. | outcome |
| 54 | Top Surgery and Chest Dysphoria Among Transmasculine and Nonbinary Adolescents and Young Adults | Ascha M, Sasson DC, Sood R, Cornelius JW, Schauer JM, Runge A, Muldoon AL, Gangopadhyay N, Simons L, Chen D, Corcoran JF, Jordan SW. | JAMA Pediatr. 2022 Nov 1;176(11):1115-1122. doi: 10.1001/jamapediatrics.2022.3424. | surgical outcomes |
| 55 | Is Social Gender Transition Associated with Mental Health Status in Children and Adolescents with Gender Dysphoria? | Morandini JS, Kelly A, de Graaf NM, Malouf P, Guerin E, Dar-Nimrod I, Carmichael P. | Arch Sex Behav. 2023 Apr;52(3):1045-1060. doi: 10.1007/s10508-023-02588-5. | outcome |
| 56 | Sex hormones, insomnia, and sleep quality: Subjective sleep in the first year of hormone use in transgender persons | Morssinkhof MWL, Wiepjes CM, Bosman BW, Kinds J, Fisher AD, Greenman Y, Kreukels BPC, T'Sjoen G, van der Werf YD, Heijer MD, Broekman BFP. | Sleep Med. 2023 Jul;107:316-326. doi: 10.1016/j.sleep.2023.04.028 | outcome |
| 57 | Expanding upon the relationship between gender-affirming hormone therapy, neural connectivity, mental health, and body image dissatisfaction | Grannis C, Mattson WI, Leibowitz SF, Nahata L, Chen D, Strang JF, Thobe H, Indyk JA, Nelson EE. | Psychoneuroendocrinology. 2023 Oct;156:106319. doi: 10.1016/j.psyneuen.2023.106319. | outcome |
| 58 | Eating disorder symptoms among transgender and gender diverse youth | Kramer R, Aarnio-Peterson CM, Conard LA, Lenz KR, Matthews A. | Clin Child Psychol Psychiatry. 2024 Jan;29(1):30-44. doi: 10.1177/13591045231184917.. | outcome |
| 59 | The risk of psychosis for transgender individuals: a Dutch national cohort study | Termorshuizen F, de Vries ALC, Wiepjes CM, Selten JP. | Psychol Med. 2023 Dec;53(16):7923-7932. doi: 10.1017/S0033291723002088. | outcome |
| 60 | Testosterone Therapy On Gender Dysphoria, Depression, And Suicidality In Transgender And Gender Diverse Individuals Seeking Masculinisation: a Randomised Controlled Trial | Nolan BJ, Zwickl S, Zajac JD, Cheung AS | Journal of the Endocrine Society, Volume 7, Issue Supplement_1, October-November 2023, bvad114.2100, https://doi.org/10.1210/jendso/bvad114.2100 | Adult |
| 61 | [Early Access to Testosterone Therapy in Transgender and Gender-Diverse Adults Seeking Masculinization: A Randomized Clinical Trial.](https://doi.org/10.1001/jamanetworkopen.2023.31919) | Nolan BJ, Zwickl S, Locke P, Zajac JD, Cheung AS | JAMA Netw Open. 2023 Sep 5;6(9):e2331919. doi: 10.1001/jamanetworkopen.2023.31919. | Adult |
| 62 | Transgender Youth Experiences with Implantable GnRH Agonists for Puberty Suppression. | Hobson BJ, Lett E, Hawkins LA, Swendiman RA, Nance ML, Dowshen NL. | Transgend Health. 2022 Aug 1;7(4):364-368. doi: 10.1089/trgh.2021.0006. | outcome |
| 63 | Well-being and suicidality among transgen- der youth after gender-affirming hormones. | Allen LR, Watson LB, Egan AM, Moser CN. | Clin Pract Pediatr Psychol. 2019;7(3):278-290. doi: 10.1037/cpp0000297. | number |
| 64 | Brain Maturation, Cognition and Voice Pattern in a Gender Dysphoria Case under Pubertal Suppression. | Schneider MA, Spritzer PM, Soll BMB, Fontanari AMV, Carneiro M, Tovar-Moll F, Costa AB, da Silva DC, Schwarz K, Anes M, Tramontina S, Lobato MIR. | Front Hum Neurosci. 2017 Nov 14;11:528. doi: 10.3389/fnhum.2017.00528. | number |
| 65 | Long-term effect of gender-affirming hormone treatment on depression and anxiety symptoms in transgender people: a prospective cohort study | Aldridge Z, Patel S, Guo B, Nixon E, Pierre Bouman W, Witcomb GL, Arcelus J. | Andrology. 2021 Nov;9(6):1808-1816. doi: 10.1111/andr.12884. | Adult |
| 66 | Cross-sex hormone therapy in trans persons is safe and effective at short-time follow-up: results from the European network for the investiga- tion of gender incongruence | Wierckx K, Van Caenegem E, Schreiner T, Haraldsen I, Fisher AD, Toye K, Kaufman JM, T'Sjoen G. | J Sex Med. 2014 Aug;11(8):1999-2011. doi: 10.1111/jsm.12571. | Adult |
| 67 | Cross-sex hormone therapy alters the serum lipid profile: a retrospective cohort study in 169 transsexuals | Ott J, Aust S, Promberger R, Huber JC, Kaufmann U. | J Sex Med. 2011 Aug;8(8):2361-9. doi: 10.1111/j.1743-6109.2011.02311.x. | Adult |
| 68 | Cross-Sex Hormone Treatment and Psychobiological Changes in Transsexual Persons: Two-Year Follow-Up Data. | Fisher AD, Castellini G, Ristori J, Casale H, Cassioli E, Sensi C, Fanni E, Amato AM, Bettini E, Mosconi M, Dèttore D, Ricca V, Maggi M. | J Clin Endocrinol Metab. 2016 Nov;101(11):4260-4269. doi: 10.1210/jc.2016-1276. | Adult |
| 69 | Self-perception of voice in transgender persons during cross-sex hormone therapy. | Bultynck C, Pas C, Defreyne J, Cosyns M, den Heijer M, T'Sjoen G. | Laryngoscope. 2017 Dec;127(12):2796-2804. doi: 10.1002/lary.26716. | Adult |
| 70 | Access to gender-affirming hormones during adolescence and mental health outcomes among transgender adults. | Turban JL, King D, Kobe J, Reisner SL, Keuroghlian AS. | PLoS One. 2022 Jan 12;17(1):e0261039. doi: | Adult |
| 71 | Psychosocial Functioning in Transgender Youth after 2 Years of Hormones. | Chen D, Berona J, Chan YM, Ehrensaft D, Garofalo R, Hidalgo MA, Rosenthal SM, Tishelman AC, Olson-Kennedy J. | N Engl J Med. 2023 Jan 19;388(3):240-250. doi: 10.1056/NEJMoa2206297. | outcome |
| 72 | Estradiol Concentrations and Wellbeing in Trans People Using Estradiol Hormone Therapy | Ginger A, Zwickl S, Angus LM, Leemaqz SY, Cook T, Wong AFQ, Cheung AS. | Transgend Health. 2024 Dec 16;9(6):484-491. doi: 10.1089/trgh.2023.0038. | outcome |
| 73 | Sublingual Estradiol Offers No Apparent Advantage Over Combined Oral Estradiol and Cyproterone Acetate for Gender-Affirming Hormone Therapy of Treatment-Naive Trans Women: Results of a Prospective Pilot Stud | Yaish I, Gindis G, Greenman Y, Moshe Y, Arbiv M, Buch A, Sofer Y, Shefer G, Tordjman K. | Transgend Health. 2023 Dec 13;8(6):485-493. doi: 10.1089/trgh.2023.0022. | outcome |

| 74 | Cross-Sex Hormones and Metabolic Parameters in Adolescents With Gender Dysphoria | Jarin J, Pine-Twaddell E, Trotman G, Stevens J, Conard LA, Tefera E, Gomez-Lobo V. | Pediatrics. 2017 May;139(5):e20163173. doi: 10.1542/peds.2016-3173. | outcome |
| --- | --- | --- | --- | --- |
| 75 | Physiologic Response to Gender-Affirming Hormones Among Transgender Youth | Olson-Kennedy J, Okonta V, Clark LF, Belzer M. | J Adolesc Health. 2018 Apr;62(4):397-401. doi: 10.1016/j.jadohealth.2017.08.005. | outcome |
| 76 | Circulating levels of follistatin from puberty to menopause | Kettel LM, DePaolo LV, Morales AJ, Apter D, Ling N, Yen SS. | Fertil Steril. 1996 Mar;65(3):472-6. | outcome |
| 77 | Safety and rapid efficacy of guideline-based gender-affirming hormone therapy: an analysis of 388 individuals diagnosed with gender dysphoria | Meyer G, Mayer M, Mondorf A, Flügel AK, Herrmann E, Bojunga J. | Eur J Endocrinol. 2020 Feb;182(2):149-156. doi: 10.1530/EJE-19-0463. | outcome |
| 78 | Determinants of Bone Mineral Density in Transgender Youth | Marwa A, Misra M, Lopez X. | Transgend Health. 2022 Jun 13;7(3):213-218. doi: 10.1089/trgh.2020.0111. | outcome |
| 79 | Proandrogenic and antiandrogenic progestins in transgender youth: differential effects on body composition and bone metabolism. | Tack LJW, Craen M, Lapauw B, Goemaere S, Toye K, Kaufman JM, Vandewalle S, T’Sjoen G, Zmierczak HG, Cools M. | Journal of Clinical Endocrinology and Metabolism 2018 103 2147–2156. | outcome |
| 80 | Low bone mass is prevalent in male-to-female transsexual persons before the start of cross-sex hormonal therapy and gonadectomy. | Van Caenegem E, Taes Y, Wierckx K, Vandewalle S, Toye K, Kaufman JM, Schreiner T, Haraldsen I & T’Sjoen G. | Bone. 2013 May;54(1):92-7. doi: 10.1016/j.bone.2013.01.039. | outcome |
| 81 | Bone safety during the first ten years of gender-affirming hormonal treatment in transwomen and transmen. | Wiepjes CM, de Jongh RT, de Blok CJ, Vlot MC, Lips P, Twisk JW, den Heijer M. | J Bone Miner Res. 2019 Mar;34(3):447-454. doi: 10.1002/jbmr.3612. | outcome |
| 82 | Bone geometry and trabecular bone score in transgender people before and after short- and long-term hormonal treatment. | Wiepjes CM, Vlot MC, de Blok CJM, Nota NM, de Jongh RT, den Heijer M. | Bone. 2019 Oct;127:280-286. doi: 10.1016/j.bone.2019.06.029. | outcome |
| 83 | Eighteen-Year Effect of Androgen Therapy on Bone Mineral Density in Trans(gender) Men | Broulik PD, Urbanek V & Libansky P. | Horm Metab Res. 2018 Feb;50(2):133-137. doi: 10.1055/s-0043-118747. | outcome |
| 84 | Low bone mineral density in early pubertal transgender/gender diverse youth: findings from the trans youth care study. | Lee JY, Finlayson C, Olson-Kennedy J, Garofalo R, Chan YM, Glidden DV, Rosenthal SM. | J Endocr Soc. 2020 Jul 2;4(9):bvaa065. doi: 10.1210/jendso/bvaa065. | outcome |
| 85 | Long-term evaluation of cross-sex hormone treatment in transsexual persons. | Wierckx K, Mueller S, Weyers S, Van Caenegem E, Roef G, Heylens G, T'Sjoen G. | Sex Med. 2012 Oct;9(10):2641-51. doi: 10.1111/j.1743-6109.2012.02876.x. | outcome |
| 86 | Bone mass, bone geometry, and body composition in female-to-male transsexual persons after long-term cross-sex hormonal therapy. | Van Caenegem E, Wierckx K, Taes Y, Dedecker D, Van de Peer F, Toye K, Kaufman JM, T'Sjoen G. | The Journal of clinical endocrinology and metabolism, 97(7), 2503–2511. | outcome |
| 87 | Cyproterone Acetate vs Leuprolide Acetate in Combination With Transdermal Oestradiol in Transwomen: A Comparison of Safety and Effectiveness. | Gava G, Cerpolini S, Martelli V, Battista G, Seracchioli R, Meriggiola MC. | Clin Endocrinol (Oxf). 2016 Aug;85(2):239-46. doi: 10.1111/cen.13050. | outcome |
| 88 | Transient Elevated Serum Prolactin in Trans Women Is Caused by Cyproterone Acetate Treatment. | Defreyne J, Nota N, Pereira C, Schreiner T, Fisher AD, den Heijer M, T'Sjoen G. | LGBT Health. 2017 Oct;4(5):328-336. doi: 10.1089/lgbt.2016.0190. | outcome |
| 89 | Subcutaneous Injection of Testosterone Is an Effective and Preferred Alternative to Intramuscular Injection: Demonstration in Female-to-Male Transgender Patients. | Spratt DI, Stewart II, Savage C, Craig W, Spack NP, Chandler DW, Spratt LV, Eimicke T, Olshan JS. | J Clin Endocrinol Metab. 2017 Jul 1;102(7):2349-2355. doi: 10.1210/jc.2017-00359. | outcome |
| 90 | Predicting successful sperm retrieval in transfeminine adolescents after testicular biopsy | Peri A, Ahler A, Gook D, O'Connell MA, Bourne H, Nightingale M, Telfer M, Jayasinghe Y, Pang KC | Assist Reprod Genet. 2021 Oct;38(10):2735-2743. doi: 10.1007/s10815-021-02293-z. | number |
| 91 | The effect of testosterone on ovulatory function in transmasculine individuals | Taub RL, Ellis SA, Neal-Perry G, Magaret AS, Prager SW, Micks EA. | Am J Obstet Gynecol. 2020 Aug;223(2):229.e1-229.e8. doi: 10.1016/j.ajog.2020.01.059. Epub 2020 Feb 8. | outcome |
| 92 | Impaired semen quality in trans women: prevalence and determinants | de Nie I, Meißner A, Kostelijk EH, Soufan AT, Voorn-de Warem IAC, den Heijer M, Huirne J, van Mello NM. | Hum Reprod. 2020 Jul 1;35(7):1529-1536. doi: 10.1093/humrep/deaa133. | outcome |
| 93 | Sexual Experiences of Young Transgender Persons During and After Gender-Affirmative Treatment | Bungener SL, de Vries ALC, Popma A, Steensma TD. | Pediatrics. 2020 Dec;146(6):e20191411. doi: 10.1542/peds.2019-1411. | outcome |
| 94 | Sperm parameters in Israeli transgender women before and after cryopreservation | Barda S, Amir H, Mizrachi Y, Dviri M, Yaish I, Greenman Y, Sofer Y, Azem F, Hauser R, Lantsberg D. | Andrology. 2023 Sep;11(6):1050-1056. doi: 10.1111/andr.13369. | Adult |
| 95 | Semen parameters among transgen- der women with a history of hormonal treat- ment. | Adeleye AJ, Reid G, Kao CN, Mok-Lin E, Smith JF. | Urology. 2019 Feb;124:136-141. doi: 10.1016/j.urology.2018.10.00 | Adult |
| 96 | Reproductive functions and fertility preservation in transgender women: a French case series. | Sermondade N, Benaloun E, Berthaut I, Moreau E, Prades M, Béranger A, Chabbert-Buffet N, Johnson N, Lévy R, Dupont C | Reprod Biomed Online. 2021 Aug;43(2):339-345. doi: 10.1016/j.rbmo.2021.04.016. | Adult |
| 97 | Fertility preservation for adolescent and young adult transmen: a case series and insights on oocyte cryopreservation. | Barrett F, Shaw J, Blakemore JK, Fino ME | Front Endocrinol (Lausanne). 2022 May 24;13:873508. doi: 10.3389/fendo.2022.873508. | outcome |
| 98 | Fertility Preservation Outcomes in Adolescent and Young Adult Feminizing Transgender Patients | Barnard EP, Dhar CP, Rothenberg SS, Menke MN, Witchel SF, Montano GT, Orwig KE, Valli-Pulaski H. | . Pediatrics. 2019 Sep;144(3):e20183943. doi: 10.1542/peds.2018-3943. | number |

| 99 | Effect of gender-affirming hormone use on coagulation profiles in transmen and transwomen | Scheres LJJ, Selier NLD, Nota NM, van Diemen JJK, Cannegieter SC, den Heijer M. | J Thromb Haemost. 2021 Apr;19(4):1029-1037. doi: 10.1111/jth.15256. | Adult |
| --- | --- | --- | --- | --- |
| 100 | Thrombosis Risk in Transgender Adolescents Receiving Gender-Affirming Hormone Therapy | Mullins ES, Geer R, Metcalf M, Piccola J, Lane A, Conard LAE, Mullins TLK. | Pediatrics. 2021 Apr;147(4):e2020023549. doi: 10.1542/peds.2020-023549.. | outcome |
| 101 | The effect of transdermal gender-affirming hormone therapy on markers of inflammation and hemostasis | Schutte MH, Kleemann R, Nota NM, Wiepjes CM, Snabel JM, T'Sjoen G, Thijs A, den Heijer M. | PLoS One. 2022 Mar 15;17(3):e0261312. doi: 10.1371/journal.pone.0261312. | Adult |
| 102 | Cardiovascular outcomes in transgender individuals in Sweden after initiation of gender-affirming hormone therapy | Karalexi MA, Frisell T, Cnattingius S, Holmberg D, Holmberg M, Kollia N, Skalkidou A, Papadopoulos FC. | Eur J Prev Cardiol. 2022 Nov 8;29(15):2017-2026. doi: 10.1093/eurjpc/zwac133. | outcome |
| 103 | The prevalence, phenotype and cardiometabolic risk of polycystic ovary syndrome in treatment-naïve transgender people assigned female at birth | Oğuz SH, Boyraz BS, Ertürk B, Yıldız BO. | Endocrine. 2024 Apr;84(1):287-292. doi: 10.1007/s12020-023-03648-5. | Adult |
| 104 | Erythrocytosis and thromboembolic events in transgender individuals receiving gender-affirming testosterone | Oakes M, Arastu A, Kato C, Somers J, Holly HD, Elstrott BK, Dy GW, Kohs TCL, Patel RR, McCarty OJT, DeLoughery TG, Milano C, Raghunathan V, Shatzel JJ. | Thromb Res. 2021 Nov;207:96-98. doi: 10.1016/j.thromres.2021.09.005. | Adult |
| 105 | Gender-affirming hormone therapy in Portugal: knowledge, safety and adherence | Santos RB, Lemos C, Saraiva M. | Int J Transgend Health. 2023 Dec 25;26(1):105-118. doi: 10.1080/26895269.2023.2296542. | outcome |
| 106 | Blood pressure effects of gender-affirming hormone therapy in transgender and gender-diverse adults. | Banks K, Kyinn M, Leemaqz SY, Sarkodie E, Goldstein D, Irwig MS. | Hypertension. 2021 Jun;77(6):2066-2074. doi: 10.1161/HYPERTENSIONAHA.120.16839. | outcome |
| 107 | Occurrence of acute cardiovascular events in trans- gender individuals receiving hormone therapy. . | Nota NM, Wiepjes CM, de Blok CJM, Gooren LJG, Kreukels BPC, den Heijer M. | Circulation. 2019 Mar 12;139(11):1461-1462. doi: 10.1161/CIRCULATIONAHA.118.038584. | outcome |
| 108 | Association of high-density lipoprotein cholester- ol with sex steroid treatment in transgender and gender-diverse youth. | Millington K, Finlayson C, Olson-Kennedy J, Garofalo R, Rosenthal SM, Chan Y-M. | JAMA Pediatr. 2021 May 1;175(5):520-521. doi: 10.1001/jamapediatrics.2020.5620 | outcome |
| 109 | Cross-sex hormones and acute cardiovascular events in transgender persons: a cohort study, | Getahun D, Nash R, Flanders WD, Baird TC, Becerra-Culqui TA, Cromwell L, Hunkeler E, Lash TL, Millman A, Quinn VP, Robinson B, Roblin D, Silverberg MJ, Safer J, Slovis J, Tangpricha V, Goodman M. | Ann Intern Med. 2018 Aug 21;169(4):205-213. doi: 10.7326/M17-2785. | outcome |
| 110 | Cardiovascular risk in Danish transgender persons: a matched historical cohort study, | Glintborg D, Rubin KH, Petersen TG, Lidegaard Ø, T'Sjoen G, Hilden M, Andersen MS. | Eur J Endocrinol. 2022 Aug 5;187(3):463-477. doi: 10.1530/EJE-22-0306. | outcome |
| 111 | Cardiovascular Disease Risk Factors and Myocardial Infarction in the Transgender Population. | Alzahrani T, Nguyen T, Ryan A, Dwairy A, McCaffrey J, Yunus R, Forgione J, Krepp J, Nagy C, Mazhari R, Reiner J. | Circ Cardiovasc Qual Outcomes. 2019 Apr;12(4):e005597. doi: 10.1161/CIRCOUTCOMES.119.005597. | outcome |
| 112 | Compromised endothelial function in transgender men taking testosterone | Gulanski BI, Flannery CA, Peter PR, Leone CA, Stachenfeld NS. | Clin Endocrinol (Oxf). 2020 Feb;92(2):138-144. doi: 10.1111/cen.14132. | Adult |
| 113 | Prevalence of cardiovascular disease and cancer during cross-sex hormone therapy in a large cohort of trans persons: a case-control study. | Prevalence of cardiovascular disease and cancer during cross-sex hormone therapy in a large cohort of trans persons: a case-control study. | Eur J Endocrinol. 2013 Sep 13;169(4):471-8. doi: 10.1530/EJE-13-0493. | outcome |
| 114 | Mental Health Concerns and Insurance Denials Among Transgender Adolescents | Nahata L, Quinn GP, Caltabellotta NM, Tishelman AC | LGBT Health. 2017 Jun;4(3):188-193. doi: 10.1089/lgbt.2016.0151 | No follow up data |
| 115 | Cross-Sex Hormone Therapy in Trans Persons Is Safe and Effective at Short-Time Follow-Up: Results From the European Network for the Investigation of Gender Incongruence. | Wierckx K, Van Caenegem E, Schreiner T, Haraldsen I, Fisher AD, Toye K, Kaufman JM, T'Sjoen G. | J Sex Med. 2014 Aug;11(8):1999-2011. doi: 10.1111/jsm.12571. | outcome |
| 116 | Lifetime Risk of Venous Thromboembolism in Two Cohort Studies. | Bell EJ, Lutsey PL, Basu S, Cushman M, Heckbert SR, Lloyd-Jones DM, Folsom AR. | Am J Med. 2016 Mar;129(3):339.e19-26. doi: 10.1016/j.amjmed.2015.10.014. | Adult |
| 117 | Effects of Cross-Sex Hormone Treatment on Cardiovascular Risk Factors in Transsexual Individuals. Experience in a Specialized Unit in Catalonia | Quirós C, Patrascioiu I, Mora M, Aranda GB, Hanzu FA, Gómez-Gil E, Godás T, Halperin I. | Endocrinol Nutr. 2015 May;62(5):210-6. doi: 10.1016/j.endonu.2015.02.001. | outcome |
| 118 | Effects of sex steroids on cardiovascular risk profile in transgender men under gender affirming hormone therapy. | Aranda G, Mora M, Hanzu FA, Vera J, Ortega E, Halperin I. | Endocrinol Diabetes Nutr (Engl Ed). 2019 Jun-Jul;66(6):385-392. English, Spanish. doi: 10.1016/j.endinu.2018.11.004. | Outcome |
| 119 | Incidence of Venous Thromboembolism in Transgender Women Receiving Oral Estradiol | Arnold JD, Sarkodie EP, Coleman ME, Goldstein DA. | J Sex Med. 2016 Nov;13(11):1773-1777. doi: 10.1016/j.jsxm.2016.09.001. . | Adult |
| 120 | No Venous Thromboembolism Increase Among Transgender Female Patients Remaining on Estrogen for Gender-Affirming Surgery. | Kozato A, Fox GWC, Yong PC, Shin SJ, Avanessian BK, Ting J, Ling Y, Karim S, Safer JD, Pang JH. | J Clin Endocrinol Metab. 2021 Mar 25;106(4):e1586-e1590. doi: 10.1210/clinem/dgaa966. | Adult |
| 121 | Safety and Rapid Efficacy of Guideline-Based Gender-Affirming Hormone Therapy: An Analysis of 388 Individuals Diagnosed With Gender Dysphoria. | Meyer G, Mayer M, Mondorf A, Flügel AK, Herrmann E, Bojunga J. | Eur J Endocrinol. 2020 Feb;182(2):149-156. doi: 10.1530/EJE-19-0463. | outcome |
| 122 | Observational Study of Hypertension and Thromboembolism Among Transgender Patients Using Gender-Affirming Hormone Therapy | Pyra M, Casimiro I, Rusie L, Ross N, Blum C, Keglovitz Baker K, Baker A, Schneider J. | Transgend Health. 2020 Mar 16;5(1):1-9. doi: 10.1089/trgh.2019.0061. | outcome |
| 123 | Prostatic Metaplasia of the Vagina and Uterine Cervix: An Androgen-associated Glandular Lesion of Surface Squamous Epithelium | Anderson WJ, Kolin DL, Neville G, Diamond DA, Crum CP, Hirsch MS, Vargas SO. | Am J Surg Pathol. 2020 Aug;44(8):1040-1049. doi: 10.1097/PAS.0000000000001486. | Adult |
| 124 | Incidence of Cancer and Premalignant Lesions in Surgical Specimens of Transgender Patients | Jacoby A, Rifkin W, Zhao LC, Bluebond-Langner R. | Plast Reconstr Surg. 2021 Jan 1;147(1):194-198. doi: 10.1097/PRS.0000000000007452. | Adult |
| 125 | Frequency and outcomes of benign breast biopsies in trans women: A nationwide cohort study | De Blok CJ, Dijkman BA, Wiepjes CM, Konings IR, Dreijerink KM, Barbé E, den Heijer M. | Breast. 2021 Jun;57:118-122. doi: 10.1016/j.breast.2021.03.007. | Adult |
| 126 | Incidence of testicular cancer in trans women using gender-affirming hormonal treatment: a nationwide cohort study | De Nie I, Wiepjes CM, de Blok CJM, van Moorselaar RJA, Pigot GLS, van der Sluis TM, Barbé E, van der Voorn P, van Mello NM, Huirne J, den Heijer M. | BJU Int. 2022 Apr;129(4):491-497. doi: 10.1111/bju.15575. | Adult |
| 127 | Cohort study of cancer risk among insured transgender people. | Silverberg MJ, Nash R, Becerra-Culqui TA, Cromwell L, Getahun D, Hunkeler E, et al. . | Ann Epidemiol. 2017 Aug;27(8):499-501. doi: 10.1016/j.annepidem.2017.07.007. | Outcome |
| 128 | Breast cancer risk in transgender people re- ceiving hormone treatment: nationwide co- hort study in the Netherlands. | de Blok CJM, Wiepjes CM, Nota NM, van Engelen K, Adank MA, Dreijerink KMA, Barbé E, Konings IRHM, den Heijer M | BMJ. 2019 May 14;365:l1652. doi: 10.1136/bmj.l1652. | outcome |
