# Supplementary material for: Use of gonadotropin-releasing hormone agonists in transgender and gender diverse youth: a systematic review
Source: Front Endocrinol (Lausanne). 2025 May 14;16:1555186. doi: 10.3389/fendo.2025.1555186 (PMC12116301; doi:10.3389/fendo.2025.1555186)
Supplement: Supplementary file 3 [file Table1.docx]

**Supplementary Table 1A.** Literature analysis after PICOS selection: summary of the studies in alphabetical order for first author and evidence grading for each study that reported physical changes and hormone levels on GnRHa treatment. *Data are expressed as mean±SD, unless otherwise stated.*

Abbreviations: AFAB: Assigned Female At Birth; AMAB: Assigned Male At Birth; AST: Aspartate Aminotransferase; BA: Bone Age; BMI: Body Mass Index; DBP: Diastolic Blood Pressure; FH: Final Height; FSH: Follicle Stimulating Hormone; GAHT: Gender Affirming Hormone Therapy; GD: Gender Dysphoria; HD: High Dose; HOMA-: Homeostatic Model Assessment; HT: Height; HV: Height Velocity; IR: Insulin Resistance; LBM: Lean Body Mass; LDL: Low-Density Lipoprotein; LH: Luteinizing Hormone; NS: Not Significant; PAH: Predicted Adult Height; PE: Physical Examination; PICOS: Population, Intervention, Comparison, Outcome, Study Design; PS: Pubertal Suppression; SBP: Systolic Blood Pressure; SD: Standard Deviation; TBF: Total Body Fat; TH: Tanner Height; TV: Testicular Volume; WC: Waist Circumference; WHR: Waist-Hip Ratio; WT: Weight

| **Study design** | **Sample, assigned sex at birth, age, follow-up duration, period (years), region and comparator** | **Methods** | **Treatment (Range of age at start and mean duration)** | **Outcomes** | **Study strenghts  Study limitations** | **Level of evidence** |
| --- | --- | --- | --- | --- | --- | --- |
| Boogers 2022^16^  Retrospective | 161 adolescents (all AMAB)  Age: <18 y  F/up: adult age (reached FH) Period: 1972-2018 Region: Netherlands (Amsterdam)  Comparator: 88 with BA <16 y at starting PS *vs* (control) 73 post-pubertal with BA ≥16 y or with FH without measurement at PS | Data from Amsterdam cohort of gender dysphoria (ACOG) database | GnRHa (triptorelin 3.75 mg every 4 wks or pamorelin 11.25 mg every 10-12 wks) Mean duration 2.4 ± 0.8 y  followed by GAHT from 15-16 y of age (if predicted FH was too high, Estrogen at high dose was proposed) | **Growth decelerated** to 3.5±1.3 cm/y during GnRHa and accelerated during GAHT **Bone maturation decreased** during GnRHa, resulting in a BA delayed by 1.6±0.8 y at the start of GAHT  FH was slightly lower than predicted at start of GnRHa, but not significantly different from TH  With regular dose Estradiol treatment: FH was 1.5±4.2 cm lower than predicted at start GAHT, but above TH by 1.1±4.5 cm  **High Dose (6 mg) and in particular EE (100-200 mcg/d orally) treatment (vs regular) reduced FH** With High dose (6mg): FH was 0.3±4.3 cm above predicted at start of GnRHa and 2.7±4.3 below predicted at start of GnRHa, and 1.3 cm below TH With growth reductive (100-200 mcg) EE treatment: FH was 4.7±4.1 cm below predicted at start of GnRHa, 4.8±3.8 cm below predicted at start of GAHT, 1.8± 7.3 cm below TH | Strengths:  Standardized treatment protocol, long f/up, focus on early puberty  Limitations:  Retrospective design, lack of a control group | ⊕⊕⊕⊖ Moderate quality evidence |
| Ciancia 2023^15^  Retrospective | 32 adolescents (10 AFAB, 22 AMAB)  Age: 11-14 y F/up: 5-6 y (reached FH) Period: 2004-2023 Region: Belgium (Ghent)  No control group | Chart review, BA assessment, TH and PAH calculations | GnRHa (triptorelin 11.25 mg every 12 wks): AFAB 12.37±0.74 to 15.97±0.46 y, AMAB from 13.10± 1.12 to 15.90±0.33 y  mean duration: AFAB 3.61±0.52 y, AMAB 2.8±1.22 y  followed by GAHT: from 15.90±0.33 y  AMAB, 15.97±0.46 y AFAB | **Early GnRHa and GAHT do not impact FH** AFAB: Total HT gain from start of GnRHa and FH: 14.62±4.08 cm (of this, 70% achieved before start of GAHT) AMAB: Total HT gain from start of GnRHa and FH: 20.68±7.66 cm (of this, 61% achieved before start of GAHT)  **AFAB and AMAB achieved a FH in line with sex assigned at birth, rather than experienced gender**  AFAB: TH for sex assigned at birth was best predictor for FH: the difference was 1.57±3.1 (p=0.168); TH for experienced gender overestimated FH: FH-TH for EG = -11.43±3.1 cm (p<0.01)  AMAB: TH for sex assigned at birth was best predictor for FH: the difference was -0.98±4.17 (p=0.319); TH for EG underestimated FH: FH-TH for EG = 12.02± 4.17 cm (p<0.01) | Strengths:  Standardized treatment protocol, long follow-up, focus on early puberty  Limitations:  Small sample size, lack of control group, retrospective design | ⊕⊕⊖⊖ Low quality evidence |
| Eitel 2023^39^  Cross-sectional | 48 adolescents (32 AMAB, 16 AFAB)    Age: 13.1±2.5 y  F/up: 6 m  Period: 2016-2021  Region: USA (Seattle)    Comparator: Eligard vs. Lupron | Retrospective chart review, hormone levels obtained 1 hour post-injection (data on n=55: 42 post Eligard,  13 post Lupron) | GnRHa (Lupron or Eligard 22.5 mg every 3 m)  Age at first injection: 13.7±2.7 | **Clinical puberty suppression in all patients, biochemical suppression was higher with Eligard** (90% vs, 69% of Lupron, p=0.06) | Strengths:  Direct comparison of Lupron and Eligard, assessment of biochemical and clinical suppression  Limitations:  Small sample size, retrospective design, potential bias due to concurrent GAHT use.  Short f/up | ⊕⊖⊖⊖ Low quality evidence |
| Fisher 2024^4^  Prospective | 36 adolescents (14 AMAB, 22 AFAB)  Age: 14.2±1.9 y  F/up: 3-12 m  Period: 2014-2020  Region: Italy (Florence)  No control group | Anthropometry, physical examination, BP,  laboratory measurements | GnRHa (triptorelin 3.75 mg every 28 days) Age range: 11-15 AMAB, 9-17 AFAB Duration: 3-12 m | **Reduction of Tanner stage (p=0.005)**  **Reduction in LH and FSH, Ferriman Gallwey score and Global Acne Grading System. In AMAB: reduction of HT percentile, T and HDL. In AFAB: increase in BMI percentile, reduction in E.** | Strenghts:  Multidisciplinary approach, longitudinal tracking of both psychological and endocrinological outcomes​  Limitations:  Small sample size, limited bone health data, lack of control group for ethical reasons​ | ⊕⊕⊕⊖ Moderate quality evidence |
| Ghelani 2020^40^  Retrospective | 36 adolescents (11 AMAB, 25 AFAB)  Age: 15-17 y F/up: 12 m Period: 2013-2015 Region: United Kingdom (London)  Comparator: pre-post | Tanita body composition analysis at 0, 6, and 12 m; measurements of HT, WT, BMI, and lean mass SDS | GnRHa (triptorelin) Age: 16.5 y (15.8–17.2) Duration: at least 1 y | Significant decrease in HT and lean mass SDS in trans girls; no significant changes in trans boys; BMI and WT SDS remained stable | Strengths: -  Limitations:  Small sample size, lack of control group, potential confounding lifestyle factors. Short f/up | ⊕⊖⊖⊖ Very low quality evidence |
| Klaver 2018^12^  Retrospective | 192 adolescents (71 AMAB, 121 AFAB)  Age: 14.5±1.8 y (AMAB) - 15.3±2.0 y (AFAB) F/up: 6-10 y (up to 22 y) Period: 1998-2014 Region: Netherlands (Amsterdam)  Comparator: reference values for age-matched peers | Data collected from medical records; Body composition measured using DXA; Linear mixed model regression to examine changes over time; SDS calculated for comparison with peers | GnRHa (triptorelin) started at 12 y: AMAB 14.5±1.8 y; AFAB 15.3±2.0 y  Duration AMAB: 2.1 (1.0-2.8) y AFAB: 1.0 (0.5-2.9) y  Followed by GAHT from 16 y of age: 16.4±1.1 y AMAB; 16.9 ± 0.9 y AFAB | **During treatment, WHR and body composition changed toward the affirmed sex**  **At 22 y of age, AMAB compared better to age-matched ciswomen than to cismen, whereas AFAB were between reference values for ciswomen and cismen**  AMAB: WC increased +8 cm (p<0.01), HT increased +17 cm (p<0.001), WHR decreased -0.04 (p<0.01), LBM decreased -9% (p < 0.001)  AFAB: WC increased +6 cm (p<0.01), HT increased +5 cm (p<0.01), WHR increased +0.03 (p<0.01), LBM increased +3% (p<0.01)  Adjustment for Tanner stage at start of treatment, or BMI at start of treatment.  % of body fat decreased in gynoid region, with no change in android region | Strengths:  Largest group of transgender adults treated from adolescence; Comprehensive data on body composition and shape changes  Limitations:  No direct control group; Retrospective design; Dietary and physical activity factors not systematically recorded | ⊕⊕⊕⊖ Moderate quality evidence |
| Klaver 2020^17^  Retrospective | 192 adolescents (71 AMAB, 121 AFAB)  Age: 15 y  F/up: up to 22 y  Period: 1998-2015  Region: Netherlands (Amsterdam)    Comparator: national or international reference data | Medical record review of anthropometry, BP,  laboratory measurements, and whole body DEXA | GnRHa (triptorelin every 4 wks), mean age of start: 14.6±1.8 y in AMAB, 15.2±2.0  y in AFAB    Median duration of GnRHa monotherapy:  AMAB 2.1 y (1.0–2.7), AFAB 1.0 y (0.5–2.9)    GAHT started at a mean age of 16.4±1.1 in AMAB, and of 16.9±0.9 y in AFAB    Median duration of GnRHa + GAHT:  AMAB 3.1 (2.5–3.6) y, AFAB 2.3 (1.8–2.8) y | Changes in BMI, total cholesterol, LDL cholesterol, HDL cholesterol, SBP, DBP, insulin, HOMA during GnRHa monotherapy and GnRHa + GAHT **are similar to those in the general adolescent population**    At age 22, **obesity prevalence was 6.6%-9.9% in AFAB and AMAB vs 2.2% in ciswomen, and 3.0% in cismen** | Strengths:  Comprehensive longitudinal data collection with anthropometric and metabolic assessments.  Limitations:  Retrospective design, lack of information regarding lifestyle. Short f/up | ⊕⊕⊕⊖ Moderate quality evidence |
| Navabi 2021^11^  Retrospective | 172 adolescents with GD (51 AMAB, 119 AFAB, 2 nonbinary)  Age: <18 y F/up: 1 y Period: 2006-2017 Region: Canada | DXA scans for BMD measurements; serum 25-hydroxyvitamin D levels assessed; body composition analysis including lean body mass and total body fat percentage | GnRHa (leuprolide acetate, 3 doses of 7.5 mg im every 4 wks, followed by 11.25 mg im every 12 wks  Age at start: n.a. Duration: n.a. | Increase in total body fat percentage; vitamin D deficiency common at baseline  An increase in gynoid (%fat), and android (%fat) in AFAB e AMAB was detected, without changes in BMI *z* score | Strengths:  Large sample size, comprehensive body composition assessment  Limitations:  Retrospective design, lack of control group, potential variability in baseline physical activity levels. Short f/up | ⊕⊕⊕⊖ Moderate quality evidence |
| Nokoff 2021^48^  Cross-sectional | 17 adolescents (8 AMAB, 9 AFAB)    Age: 13.7±1.2 y (AMAB) - 13.8±1.7 y (AFAB)  F/up: ≥3 m  Period: 2016-2019  Region: USA (Colorado)    Comparator:  14 cisgender females (13.9±1.7 y) and 17 cisgender males (13.9±0.9 y) | Medical record review of clinical and metabolic outcomes, total body DEXA | GnRHa,  age of start: 12.8±1.3 y (AMAB) 12.1±1.9 y (AFAB); mean duration:  11.3±7 m (AMAB); 20.9±19.8 m (AFAB), | **AMAB and AFAB have lower estimated insulin sensitivity and higher glycemic markers and body fat than cisgender controls with similar characteristics**    AMAB had higher HOMA-IR (p=0.04), HbA1c (p=0.01), AST (p=0.01), leptin (p<0.01) and % of body fat (p<0.01), and  lower insulin sensitivity (1/fasting insulin, p=0.03), total testosterone (p<0.01), SBP (p<0.01) and % of lean mass (p<0.01) vs cisgender males.    AFAB had higher HOMA-IR (p=0.01), fasting glucose (p=0.01), HbA1C (p=0.04), leptin (p<0.01), AST (p=0.02), and % of body fat (p=0.04) and lower insulin sensitivity (p=0.03), total estradiol (p=0.01) and total lean mass (32.3±5.2 vs. 36.4±7.8 kg, p=0.01) vs cisgender females. | Strengths:  Comparison of transgender youth with cisgender controls, detailed assessment of insulin sensitivity and body composition  Limitations:  Cross-sectional design, small sample size  Wide range of f/up    Patients matched on age, without adjustment for pubertal stage and/or physical activity level | ⊕⊖⊖⊖ Very low quality evidence |
| Olson-Kennedy  2021^49^  Retrospective | 66 adolescents  (32 AMAB, 34 AFAB)    Age at start: 11.8 y (AMAB), 10.8 y (AFAB)  F/up: 2-12 m  Period: 2006-2016  Region: USA (Los Angeles, Boston, Chicago, San Francisco)    No control group | Baseline and f/up measurements of testosterone/estradiol and gonadotropins | Histrelin implants (Vantas 30.3%, SupprelinLa 69.7%)  range of age at start: AMAB 10-15 y (84% Tanner stage 2); AFAB 9-15 y (71% Tanner stage 2)  Duration: 2-12 m | **Gonadotropin and sex steroid levels were significantly decreased at T1** (p<0.01), with no differences between implants. | Strengths:  Comparison of two GnRHa implants, multicenter data collection  Limitations:  Small sample size, retrospective design. Short f/up | ⊕⊕⊖⊖ Low quality evidence |
| Pine-Twaddel 2023^52^  Retrospective | 49 adolescents (42 with GD, 7 with CPP)  Age: 11.6±2.4 y F/up: up to 71 m Period: 2010-2020 Region: USA (Baltimore/San Diego)  Comparators: GD on histrelin alone; GD on histrelin+GAHT; GD on histrelin+GAHT with irregular follow-up; CPP on histrelin | PS assessed clinically and biochemically; random hormone levels measured; Tanner staging and laboratory f/up | Histrelin implant (HI) use extended beyond 12 m; mean duration 37.5 m | Sustained biochemical and clinical pubertal suppression in most subjects; pubertal escape occurred in 16% at 15-65 m | Strengths:  Long f/up period  Limitations: Sample size, Retrospective design, non-standardized testing intervals | ⊕⊕⊖⊖ Low quality evidence |
| Schagen  2018^13^  Prospective | 127 adolescents  (73 AMAB, 54 AFAB)    Age at start: 14±1.6 y (AMAB)  14.3±2.0 y (AFAB)  F/up: 6 m - 4 y  Period: 2008-2016  Region: Netherlands (Amsterdam)    No control group | Blood tests for  DHEAS and androstenedione every 3-6 m | GnRHa (triptorelin 3.75 mg at 0, 2, and 4 wks, then every 4 wks)  range of age at start: AMAB 11.6-17.9 y; AFAB 11.5-18.6 y; duration: ~2 y    followed by GAHT (oral estradiol/intramuscular testosterone), range of age at start: AMAB 11.6-17.9 y; AFAB, 11.5-18.6; duration: ~2 y | **DHEAS levels did not significantly change during treatment in either group. In AFAB, androstenedione levels decreased during GnRHa treatment and increased during GAHT, potentially due to testosterone conversion​**    During GnRHa: AFAB: DHEAS levels rose (normal increase during adolescence) and **androstenedione decreased** during the first y (reduced ovarian androstenedione synthesis) AMAB: No change in DHEAS and androstenedione  During GAHT:  AFAB: No change in DHEAS; androstenedione rose during the first y possibly due to conversion of administered testosterone  AMAB: No change in DHEAS and androstenedione | Strenghts:  Large cohort, standardized treatment protocol, detailed f/up  Limitations:  Lack of a control group, no assessment of cortisol. Wide range of f/up | ⊕⊕⊕⊖ Moderate quality evidence |
| Schagen 2016^10^  Prospective | 116 adolescents (49 AMAB, 67 AFAB)    Age (median): 13.6 y (AMAB) -14.2 y (AFAB)  F/up: 3 m - 3 y  Period: 1998 - 2009  Region: Netherlands (Amsterdam)    No control group | Physical exams every 3 m  Blood tests for hormones and liver/renal function every 3-6 m  DXA for body composition | GnRHa (triptorelin 3.75 mg at 0, 2, and 4 wks, then every 4 wks)  range of age at start: AMAB 11.6-17.9 y, AFAB 11.1-18.6 y; for at least 3 m; mean  Duration not specified | **Significant decreases in HT SDS, testicular volume/breast volume and hormonal levels; lean body mass percentage decreased while fat percentage increased​**    HT SDS decreased in AMAB and AFAB (p<0.01) at 1 and 2 y  BMI increased in AFAB at 1 y (p=0.01)    Reduced TV in 43/49 AMAB at 1 y (p< 0.01) Regression of breast from B2 to B1    LH, FSH, T in AMAB, E2 in AFAB: suppressed within 3 m (p<0.01) ALP decreased (p< 0.01)  Lean body mass % decreased and fat increased at 1 y in AMAB and AFAB (p< 0.01) | Strenghts:  Prospective study; large number of participants; detailed monitoring and reliable measurements; comprehensive anthropometric and biochemical data  Limitations:  No control group; observational design; small sample size of adolescents in early puberty stage. Wide range of f/up | ⊕⊕⊕⊖ Moderate quality evidence |
| Schulmeister 2022^18^  Prospective | 55 adolescents (26 AMAB, 29 AFAB)    Age: 11.9 ± 1.1 y (AMAB), 11.1 ± 1.2 y (AFAB)  F/up 10-14 m  Period: 2016-2018  Region: USA (multi-site)    Comparator:  226 pre-pubertal cisgender controls (aged 11.0 ± 2.8 y) from the Bone Mineral Density in Childhood Study (BMDCS) | Anthropometric and laboratory data collection from medical records, Tanner stage assessment | GnRH analogue (84% implantable histrelin, 16% injectable leuprolide)  Range of age start: 9.0-14.5 y;  Mean duration: 12 m | No difference in the HV between AMAB (5.4 cm/y [4.2–5.7]) and AFAB (4.8 cm/y [3.5–5.5]) (p=0.2)    HV stratified by Tanner stage was comparable between transgenders and cisgenders controls    **Later Tanner stage at GnRHa initiation was associated with lower HV (p<0.01).** Controlled for age: no difference in mean HV vs prepubertal youth;  Stratified by Tanner stage, individuals starting GnRHa at Tanner stage IV had an HV (1.6 cm/y [1.5-2.9]) below that of prepubertal youth (6.1 cm/y [4.3-6.5]) (p<0.01).  BMI z-score: no difference in baseline and 12-m between AMAB and AFAB  Tanner stage at GnRHa start (p<0.01), baseline LH (p=0.04); and age at GnRHa start (p=0.02) were negatively correlated with HV after starting GnRHa.  Baseline BMI and sex hormone concentrations did not correlate with HV | Strengths:  Multi-site study, comparison with cisgender controls  Limitations:  Small sample size,  no data on BA or pretreatment HV, limited predictive power for adult HT. Short f/up | ⊕⊕⊕⊖ Moderate quality evidence |
| Valentine 2022^23^  Cross-sectional | 4.172 adolescents (2766 listed as female in chart, 1407 listed as male in chart)  Age: 9.8-10 y  F/up: 6.1 y  Period: 2009-2019  Region: USA (multisite)  Comparator: 16.453 controls | Electronic health record data | GnRHa alone 267 (6.4%); GnRH + testosterone 106 (2.5%); GnRHa + estradiol 125 (3.0%); testosterone only 832 (19.9%); estradiol only 349 (8.4%) Age at start: n.a. Mean duration: n.a. | Adolescents with GD had higher odds of overweight/obesity than controls.  Estradiol and **GnRHa alone were not associated with greater odds of cardiometabolic-related diagnoses.**  Those treated with testosterone (alone or in combination with GnRHa) had higher odds of dyslipidemia and liver dysfunction, those with testosterone alone had higher odds of overweight/obesity and hypertension. | Strengths:  Multisite data, large sample size, comparison with matched controls  Limitations:  Retrospective, cross-sectional evaluation | ⊕⊕⊕⊖ Moderate quality evidence |
| Van de Grift 2020^19^    Retrospective | 200 adolescents (66 AMAB;134 AFAB)  Age: <18 y at starting PS, >18 y at data collection (mean age at f/up: 23 y, SD = 2.9) F/up: 6-12 y  Period: 2006-2013 Region: Netherlands (Amsterdam)  Comparator: 100 controls who did not receive PS but underwent GAHT and surgery | Local registries and routine evaluations | Early-initiated GnRHa (Tanner 2/3): 43 subjects: (26 AMAB, 17 AFAB); mean age at start: 12±0.1 y; mean duration before GAHT: 3 y  Later-initiated GnRHa (Tanner 4/5): 157 subjects (40 AMAB, 117 AFAB); mean age at start: 15±0.1 y; mean duration before GAHT: 2 yr  GAHT initiated after GnRHa: early GnRHa 15±0.1 y; later GnRHa 17±1.0 y | Final HT, WT and BMI SDS not statistically different among the 3 groups (early, later, no GnRHa)  AMAB: in those using GnRHa shorter penile length (p<0.01) and increased likelihood of intestinal vaginoplasty (OR 9.8)  AFAB: less breast development in early GnRHa (p<0.01), increased likelihood of not needing mastectomy (p<0.01) | Strengths:  Comprehensive evaluation of physical and surgical outcomes  Limitations:  Retrospective design | ⊕⊕⊕⊖ Moderate quality evidence |
| Willemsen 2023^34^  Retrospective | 146 adolescents (all AFAB) with GnRHa <16 y, testosterone for >6 m, reached 18 y of age  Age: 14.1 F/up: mean 3.1 y for GnRHa and 2 y for GAHT Period: 1972-2018 Region: Netherlands (Amsterdam) | Measurement of growth velocity, BA, AH, and comparison with predicted FH and midparental HT using DXA and other standardized methods | GnRHa and testosterone treatment; GnRHa start age: <16 y, followed by testosterone (6 m minimum)  Duration of PS: 3.1±0.9 y | During PS, growth velocity decreased, followed by catch-up growth during GAHT; adult HT exceeded predicted FH by 3.0 ± 3.6 cm and midparental HT by 3.9 ± 6.0 cm | Strengths:  Large cohort, detailed growth assessment, comparison with predicted FH and midparental HT  Limitations:  Retrospective design, variability in hormone regimens, potential biases in BA assessment. | ⊕⊕⊕⊖ Moderate quality evidence |
