# Supplementary material for: Use of gonadotropin-releasing hormone agonists in transgender and gender diverse youth: a systematic review
Source: Front Endocrinol (Lausanne). 2025 May 14;16:1555186. doi: 10.3389/fendo.2025.1555186 (PMC12116301; doi:10.3389/fendo.2025.1555186)
Supplement: Supplementary file 4 [file Table2.docx]

**Supplementary Table 1B.** Literature analysis after PICOS selection: summary of the studies and evidence grading for each study that reported bone outcomes of GnRHa treatment. *Data are expressed as mean ± SD, unless otherwise stated. p1 start GnRHa-start GAH; p2 start GAH-24 m after GAH.*

Abbreviations: BA: Bone Age; BMAD: Bone Mineral Apparent Density; BMD: Bone Mineral Density; BMI: Body Mass Index; DEXA: Dual-Energy X-ray Absorptiometry; ED: Eating Disorder; EE: Ethinyl Estradiol; FN: Femoral Neck; GAH: Gender Affirming Hormone; GAH-24: Gender Affirming Hormone at 24 months; HAS: Height Age Standard Deviation; HAZ: Height-for-Age Z-score; HT: Height; LS: Lumbar Spine; NS: Not Significant; PICOS: Population, Intervention, Comparison, Outcome, Study Design; PS: Pubertal Suppression; SD: Standard Deviation; SPW: Spine Width; TH: Tanner Height; WT: Weight

| **Study design** | **Sample, gender identity, age, follow-up duration, period (years), region and comparator** | **Methods** | **Treatment (Range of age at start and/or duration)** | **Outcomes** | **Study strengths - Study limitations** | **Level of evidence** |
| --- | --- | --- | --- | --- | --- | --- |
| Boogers 2023^37^  Retrospective | 87 AMAB  Age at start GnRHa  13.5±1.2  Age at GAHT:  15.8 +/-0.9 y  F/up: about 4.5 y  Period:  1972-2018  Region: Netherlands (Amsterdam)  Comparator: Divided in  3 groups:  Group 1:  GnRHa + Regular dose GAHT  Group2:  GnRHa + High dose GAHT  Group3:  GnRHa + EE | DXA at start of GnRHa, GAHT and 2 y after | GnRHa+GAHT  Duration of GnRHa:  2.3 ±0.7 y  Duration GAHT  2y  GAHT protocol  Group1:  Regular dose:  estradiol gradually increased to 2 mg  Group 2:  High dose: estradiol increased to 6 mg  Group 3:  EE (ethinyl estradiol)  100-200 µg  6 | **At baseline and before start GAHT:**  **BMD z-score at LS lower in group 2 compared to group 1.**  **BMD z-score at hip comparable in all 3 groups.**  After 2 y of GAHT:  BMD LS z-scores show a small increase in group 1 (0.13, 95%CI -0.2—0.29), while a greater increase was registered in group 2 and 3, respectively 0.42 (95%CI 0.13-0.72) and 0.68 (95%CI 0.20-1.15).  In both group 2 and 3 z-scores approached baseline values.  BMD femoral region z-score:  restoration to baseline values in all groups except for Z-score of femoral neck which remained lower than at baseline in group 1.  **Higher estrogen dosage is associated with a greater increase in LS-BMD Z-scores** Increasing dosage up to 2 mg estradiol is insufficient to optimize BMD and approximately **4 mg may be required** for adequate serum concentrations | Strengths  Limitations  No control group | ⊕⊕⊖⊖  Low quality evidence |
| Carmichael 2021^21^  Prospective | 44 adolescents (19 AFAB; 25 AMAB)   Age  12.8-14.6 y (mean 13.6)  F/up: 12-36 m  Period:  2011-2014  Region: United Kingdom (London)  No control group | DXA; serum 25-OH-vitamin D levels; | GnRHa median duration  31 m (20-42)  GAHT  Evaluations: T0, after 12m, after 24m and 36m from the start of GnRHa | There was **no change from baseline in LS BMD** at 12 m nor in **hip** BMD at 24 and 36 m, but at 24 m LS BMC and BMD were higher than at baseline (BMC +6.0 (95% CI: 4.0, 7.9); BMD +0.05 (0.03, 0.07)).  aBMD LS z-score: from baseline to 24 m: −0.5 (−1.1, 0.0) at start GnRHa, to −1.5 (−2.1, −0.8) p-values not available  aBMD hip z-score: from baseline to 24-36 m; aBMD-hip −0.5 (−1.1, 0.1) at start GnRHa; −1.4 (−2.0, −0.9)  p-values not available | Strengths:  Prospective  Limitations:  Small sample size,  No control group | ⊕⊕⊕⊖  Moderate quality evidence |
| Joseph 2019^41^  Retrospective | 70 adolescents (31 AMAB, 39 AFAB)  Age at start GnRHa:  13.0±1.1 y (AMAB), 12.9±3.0 y (AFAB)  F/up: 1 y  Period:  2011-2016  Region: United Kingdom (London)  Comparator: pre-post | DXA at start of GnRHa and annually | GnRHa  GnRHa duration  1 y in 39 subjects; 2 y in 31 subjects | **Significant change in BMD and BMAD z-scores over 2 y**  At baseline AFAB had lower BMD measures than AMAB  AFAB  Lumbar region (LS) Z-scores:  aBMD-LS -0.40± 1.43 at PS; -1.28 ±1.41 one y later (p<0.01)  BMAD-LS -0.19±1.23 at PS; -0.54± 1.40 one y later (p<0.01)  Femoral region (hip) Z-score  aBMAD-Hip -0.86±1.22 at PS; -1.44± 1.08 one y later (p<0.01)  AMAB  Lumbar region (LS) Z-scores: aBMD-LS -0.02±1.11 at PS; -0.46 ±1.12 one y later (p<0.01)  BMAD-LS 0.86± 0.15 at PS; -0.23 ±1.03 one y later (p<0.01)  Femoral region (hip) Z-score:aBMAD-Hip 0.16 ±0.91 at PS; -0.34 ±0.82 one y later (p<0.01) | Strengths  Well-defined f/up intervals  Limitations  Small sample, no control group, short f/up | ⊕⊕⊖⊖  Low quality evidence |
| Klink 2015^14^  Prospective | 34 adolescents (15 AMAB, 19 AFAB)  Age at start:  14.9 ± 1.9 y  (AMAB), 15.0 ±2.0 y (AFAB)  F/up: until 22 y  Period:  1998-2012  Region: Netherlands (Amsterdam)  Comparator: pre-post | DXA at start of GnRHa, start of GAHT, and age 22 | GnRHa + GAHT  GnRHa median duration  AFAB 1.5 y (0.25-2.5)  AMAB 1.3 y (0.5-3.8)  GAHT median duration  AFAB 5.4 y (2.8-7.8)  AMAB 5.8 y (3.0-8.0) | **In AFAB: increasing trend in BMD z-scores after GAHT**  **In AMAB: persistently low BMD z-scores, worsened by treatment**  AFAB  **LS z-score**  aBMD 0.17 ± 1.18 at PS; -0.72±0.99 at GAHT; -0.33 ±1.12 at 22 yrs; PS-GAHT p <0.001; GAHT-age 22: NS  BMAD 0.28±0.90 at PS; -0.50±0.81 at GAHT; 0.03±0.74 at 22;  p1 0.004; p2 0.002  **Femoral region Z-score**   aBMAD 0.36±0.88 at PS; -0.35±0.79 at GAHT; -0.35±0.74 at 22  p1: 0.001; p2 0.006  BMAD 0.01±0.70 at PS; -0.28±0.74 at GAHT; NA at 22yrs  p1 NS; p2 NA  AMAB  **LS z-score**  aBMD -0.77 ±0.89 at PS; -1.01±0.98 at GAHT; -1.36 ±0.83 at 22 yrs  p1NS; p2 NS  BMAD -0.44±1.10 at PS; -0.90± 0.80 at GAHT; -0.78±1.03 at 22;  p1 NS; p2 NS  **Femoral region z-score**  aBMAD -0.66±0.77 at PS; -0.95±0.63 at GAHT; -0.69±0.74 at 22  p1 NS; p2 NS  BMAD -0.93±1.22 at PS; -1.57±1.74 at GAHT; NA at 22yrs  p1 NS; p2 NA | Strengths Prospective  Consistent f/up  Limitations  Small sample size, no control group,  possible  confounders due to long f/up | ⊕⊕⊖⊖  Low quality evidence |
| Navabi 2021^11^  Retrospective | 172 adolescents with GD (51 AMAB, 119 AFAB, 2 nonbinary)  Age: <18 y F/up: 1 y Period: 2006-2017 Region: Canada  Comparator: before and after GnRHa | DXA and 25-OH-vitamin D at start of GnRHa | GnRHa  Age at start: n.a.  Duration: n.a. | At baseline **AMAB had lower BMD-LS and hip z scores, compared to AFAB.**  Vitamin D was sufficient only in 44.7% of TGD adolescents, and baseline vitamin D status was associated with BMD and BMAD z-scores at LS and hip,  **A significant decrease in BMD Ls and hip z-score was registered during GnRHa monotherapy in both AMAB and AFAB. BMAD LS z-score decreased significantly only in AFAB.** | Strengths:  Limitations:  no control group, short f/up | ⊕⊕⊖⊖  Low quality evidence |
| Schagen 2020^27^  Prospective | 121 adolescents (51 AMAB, 70 AFAB, divided among early and late-pubertal)  F/up: 3 y  Age: 12-18 y  Period:  1998-2009  Region: The Netherland  Comparator group: early vs late pubertal | DXA at start of GnRHa and yearly  + markers of bone formation(  P1NP, P3NP, osteocalcin)  and markers of bone resorption (1CTP) | GnRHa+GAHT  Age at GnRHa:  AMAB  14.1±1.7  AFAB  14.5±2.0  Duration GnRHa  AMAB  2.0±0.94 y  AFAB  1.8±1.1 y  Age at GAHT:  AMAB  16.2±1.2  AFAB  16.9±1.1 | **BMD and BMAD LS z-scores significantly decreased during 24 m of GnRHa treatment in all group, and increased significantly after 36 m of GAHT except in AMAB late pubertal (BMD-LS z-score).**  **BMD and BMAD hip z-score significantly decreased during 24 m of GnRHa treatment in all group, and increased significantly after 36 m of GAHT except in AMAB late pubertal (BMD-hip z-score) and AFAB late pubertal (BMAD hip z-score).**  **AFAB had normal z-scores at baseline and after 36 m of GAHT following GnRHa.**  **AMAB had relatively low z-scores, both at baseline and after 36 m of GAHT following GnRHa.**  Bone markers:  in all AMAB and in early pubertal AFAB significantly decreased during the treatment. | Strengths: Prospective, comprehensive bone assessments  Limitations:  no control group | ⊕⊕⊕⊖  Moderate quality evidence |
| Stoffers 2019^55^  Retrospective | 62 AFAB  Age at start GnRHa  16.5 y (11.8-18.0)  89% Tanner 4-5  F/up: 8 m  Period:  2010-2018  Region: Netherlands (Leiden)  Comparator: pre-post | DXA at start of GnRHa and at start GAHT | GnRHa+GAHT  GnRHa duration  minimum of 6 m, median duration 8 m (3-39)  GAHT  GAHT median duration  **12 m** (5-33) | **Significant decrease in BMD Z-score at LS and femoral region during GnRHa, followed by increase after GAHT although baseline values not restored.**  aBMD-LS Z-score:  0.02 ± 1.00 at start GnRHa, -0.81 ±1.02 at start GAHT; -0.66 ± 0.81 after 12 m of T  p1 <0.001; p2 <0.05  aBMD-hip Z-score:  -0.19 ± 1.04 at start GnRHa, -1.07 ±0.85 at start GAHT, -0.93 ± 0.63 12 m after T  p1p<0.001; p2<0.05 | Strengths  Limitations  Small cohort, no control group  Short f/up  Main focus on the effect of testosterone | ⊕⊕⊖⊖  Low quality evidence |
| Van der Loos 2021^57^  Retrospective | 322 youth (106 AMAB, 216 AFAB)  Age: 12-18 y  F/up: n.a.  Period:  1987-2018  Region: Netherlands  Comparator:  3 groups subdivided per pubertal stage at the start of GnRHa:  Early-mid-late puberty | DXA and  hip structure software analysis (HAS) | **GnRHa+GAHT**  **GnRHa**  Age at start: range 11.8- 16.8 y  Duration:  range 0.6-4.1 y  **GAHT**  Age at start:  range 15.3-17.5 y  Duration:  range 2.7-6.3 y | **Hip geometry (**determined by the measurement of subperiosteal width and endocortical diameter) is sexually dimorphic.  **The start of GnRHa during early puberty allows to redefine hip geometry resembling that of the experienced gender; if GnRHa are started in mid/late puberty, transgender adolescents maintaina hip geometry in line with gender assigned at birth** | Strengths:  Large cohort, comprehensive bone geometry assessment  Limitations:  no control group,  Standardized data set on HAS parameters as reference data was not available  Technical limitations inherent to measuring bone geometry with DXA | ⊕⊕⊖⊖  Low quality evidence |
| Van der Loos 2023^20^  Retrospective | 75 adolescents (25 AMAB, 50 AFAB)  Age at start GnRHa  AMAB 14.5 y  (13.4-15.7)  AFAB 14.9 y  (13.0-16.4)  Age at start GAHT  -AMAB 16.0 y  (16.0-16.8)  -AFAB 14.9 y  (16.1-17.6)  F/up 15 y  Period:  2001-2018  Region: Netherlands  Comparator: short vs. long f/up | DXA before GnRHa, at start of GAHT, at median age of 22 and of 28 y | **GnRHa** +GAHT  **At short-term GAHT:**  AMAB: 22.1 y  (21.7-22.6y)  AFAB 22.1 y  (21.9-22.5 y)  **At long-term GAHT:**  AMAB: 28.2 y  (27.0-30.8y)  AFAB 28.2 y  (26.6-30.6 y)  Mean duration  -AMAB 1.5 y (0.7-2.6y)  -AFAB 1.5y (0.7-3.1y) | **BMD-LS Z-score in AMAB decreased during GnRHa while remaining stable during GAHT. Pre-treatment levels not restored.**  **In AFAB a decrease was registered during GnRHa but increased after GAHT, with restoration of baseline values.**  **BMD-hip z-score decreased during GnRHa in both groups, but pretreatment levels were restored after GAHT.**  AMAB BMD LS z-score:  Decrease at long-term f/up (-0.87; 95% CI -1.15; -0.59 compared to start GnRHa)  AFAB BMD LS z-score:  Change from start GnRHa (0.09; 95% CI -0.09; -0.27 compared to start GnRHa) | Strengths:  Long-term f/up comprehensive BMD assessment  Limitations:  No control group  potential confounding factors due to long f/up  DXA data not available for all participants | ⊕⊕⊕⊖  Moderate quality evidence |
| Vlot 2017^22^  Retrospective | 56 adolescents (22 AMAB, 34 AFAB)  F/up: 24 m after start GAHT  Period: NA  Region: Netherlands (Amsterdam)  Comparator: pre-post | DXA, serum bone markers | GnRHa+GAHT  GnRHa mean duration  1.2 y AFAB; 1.5 y AMAB  GAHT added in incremental doses from the age of 16 y | **During GnrHa, BMAD Z-scores (mainly at LS) decreased, especially in the young (BA<14 y) AMAB group.  After 24 m of GAHT, Z-scores partially restored, especially for the LS.**  Young AMAB group  BMAD LS z-score -0.2 start GnRHa, -1.52 start GAH, -1.10 +24 m  p1<0.01; p2<0.05  **P1NP and 1CTP decreased during GnRHa treatment, indicating decreased bone turnover.  After 24 m of GAHT**, **P1NP and ICTP further decreased in all groups, except for the old (BA>14 y) AFAB.**  Old AFAB group  P1NP (median) 110 start GnRHa, 127 start GAH, 101 +24 m  p1 NS; p2<0.01  ICTP (median) 7 start GnRHa, 6.9 start GAH, 8.2 +24 m  p1 NS; p2<0.1 | Strengths  Well-defined f/up intervals, comprehensive data  Limitations  Small sample size, no control group | ⊕⊕⊕⊖  Moderate quality evidence |
