# Supplementary material for: Use of gonadotropin-releasing hormone agonists in transgender and gender diverse youth: a systematic review
Source: Front Endocrinol (Lausanne). 2025 May 14;16:1555186. doi: 10.3389/fendo.2025.1555186 (PMC12116301; doi:10.3389/fendo.2025.1555186)
Supplement: Supplementary file 5 [file Table3.docx]

**Supplementary Table 1C**. Literature analysis after PICOS selection: summary of the studies in alphabetical order for first author and evidence grading for each study that reported mental health outcomes of GnRHa treatment. *Data are expressed as mean±SD, unless otherwise stated.*

Abbreviations: ABCL: Adult Behavior Checklist; AFAB: Assigned Female At Birth; AMAB: Assigned Male At Birth; BDI: Beck Depression Inventory; BIS: Body Image Scale; CBCL: Child Behavior Checklist; CGAS: Children’s Global Assessment Scale; GAD-7: Generalized Anxiety Disorder 7-item scale; GAHT: Gender Affirming Hormone Therapy; GD: Gender Dysphoria; GIQA: Gender Identity/Gender Queer Assessment; NB: Non-Binary; NS: Not Significant; PHQ-9: Patient Health Questionnaire-9; PICOS: Population, Intervention, Comparison, Outcome, Study Design; PS: Pubertal Suppression; QID: Quick Inventory of Depressive Symptomatology; SCARED: Screen for Child Anxiety Related Disorders; SD: Standard Deviation; SDQ: Strengths and Difficulties Questionnaire; SHS: Subjective Happiness Scale; SRS-2: Social Responsiveness Scale, Second Edition; STAI: State-Trait Anxiety Inventory; SWLS: Satisfaction With Life Scale; UGDS: Utrecht Gender Dysphoria Scale; WAIS: Wechsler Adult Intelligence Scale; WISC: Wechsler Intelligence Scale for Children; YSR: Youth Self Report

| **Study design** | **Sample, gender identity, age, follow-up duration, period (years), region and comparator** | **Methods** | **Treatment (Range of age at start and mean duration)** | **Outcomes** | **Study strengths  Study limitations** | **Level of evidence** |
| --- | --- | --- | --- | --- | --- | --- |
| Achille 2020^35^  Prospective | 50 adolescents (17 AMAB, 33 AFAB)  Age: 16.2±2.2 y  F/up: 18 m  Period: 2013-2018  Region: USA    Comparator: No control group | Psychological and behavioral assessments through questionnaires (CESD-R, PHQ-9, QLES-Q-SF; Suicidal Ideation) | GnRHa (46%), GAHT (70%), both (22%)  Age 15.5±1.6 y  Duration: 18 m  Evaluations every 6 m | PS and/or GAHT improved mental health: Depression: CESD-R score: from 21.4 at baseline to 13.9 - less than 16 means no depression (*p* <0.01); PHQ-9 decreased over time (*p* <0.01)    QoL scores improved (*p* =0.09)  Suicidal ideation decreased over time across all groups vs baseline | Strengths:  Prospective, comprehensive assessment of mental health and QoL; multiple time points  of f/up  Limitations:  sample size; lack of control group | ⊕⊕⊕⊖ Moderate quality evidence |
| Arnoldussen 2022^28^  Retrospective | 72 adolescents (27 AMAB, 45 AFAB)    Age:  - At pre treatment 12.78±1.48 y;  - at evaluation at 20.40±1.03 y  F/up 2.4 y  Period: not specified  Region: Netherlands (Amsterdam)    Comparator: pre-post | Psychological and behavioral assessments through questionnaires (WISC or WAIS, CBCL, YSR) educational achievement evaluated post-treatment | GnRHa range of age at start 11.47–16.99 y; mean duration 2.40 y    followed by GAHT and gender-affirming surgery | Educational achievement was evaluated after GAHT (mean age 20.40 y)  The IQ scores and educational achievement were not significantly different between AMAB and AFAB. The mean total IQ of the participants was 100.29 (SD = 15.07). For verbal IQ, the mean was 99.53 (SD = 15.01), for performance IQ the mean was 100.72 (SD = 14.26)    Pre-treatment IQ and educational achievement after PS and GAHT were strongly associated in adolescents with GD (Nagelkerke R = 0.71)  Gender was not found to be an effect modifier in the association between total, verbal, performance IQ and educational achievement. | Strengths:  Long-term f/up, comprehensive assessment of cognitive and educational outcomes.  Limitations:  Lack of control group, only two measurement points | ⊕⊕⊕⊖ Moderate quality evidence |
| Becker 2018^31^  Retrospective cross-sectional | 82 adolescents with GD (20 AMAB, 62 AFAB)  Age: 14-21 y  F/up: unavailable Period: 2013-2015 Region: Germany  Comparator: 11 GAHT, 14 GAHT+ surgery | Psychological and behavioral assessments through questionnaires (Body Image Questionnaire) | GnRHa (n=13) Treatment duration mean 21.4 m | In participants, at baseline, both psychological functioning and QoL scores were significantly below the norm mean for all intervention groups. | Strengths:  Control group  Limitations:  Cross-sectional design Small sample size of subjects with GD receiving PS | ⊕⊖⊖⊖ Very low quality evidence |
| Becker-Hebly 2021^36^  Prospective | 75 adolescents and young adults out of 204 (11 AMAB, 64 AFAB)    Age: baseline 15.6 y, f/up: 17.4 y F/up: around 2 y  Period: 2013-2018  Region: Germany (Hamburg)    Comparator: pre-post | Psychological and behavioral assessments through questionnaires (YSR, ASR, CGAS, Kidscreen-27 and SF-8) | GnRHa, range of age at start 15.56±1.85 y, mean duration 21.4 m    followed by GAHT and/or surgery | At baseline, both psychological functioning and QoL scores were significantly below the norm mean for all intervention groups.    At f/up, adolescents who underwent PS or  GA (hormonal and surgical) interventions showed better  scores in some emotional and behavioral dimensions and physical QoL scores were like the German norm mean. For the group receiving no medical interventions, the scores still indicated psychological  impairment.    In the group receiving PS, the externalizing  problems (YSR/ASR) score and mental and physical  health-related QoL scores (Kidscreen/SF-8)  were within the norm.    However, some of the psychosocial health outcome scores were still significantly different from the norm: baseline difficulties, as reflected in the total problem score and internalizing problems score, persisted throughout the f/up period in the PS group | Strengths:  Comprehensive use of standardized instruments, Prospective  Limitations:  High drop-out rate, lack of control group, overrepresentation of AFAB individuals (85%). | ⊕⊕⊖⊖  Low quality evidence |
| Cantu 2020^33^  Prospective | 80 youth (15 AMAB, 58 AFAB, 7 NB)    Age: mean 15.1 (SD 1.8) y  F/up: 1-11 m  Period: 2017-2019  Region: USA (Portland)    Comparator: pre-post | Psychological and behavioral assessments through questionnaires (PHQ-9, GAD-7) | GnRHa (n=17) between visits, GAHT (n=28) between visits, GAHT (n=1) before the initial visit  mean age at start 15.1 (SD 1.8) y; f/up: 4.7 m | No change in depression, anxiety, suicidal ideation. Changes probably take longer to occur.  Almost all (96%) were not on GnRHa nor GAHT at initial visit.    Depression: PHQ-9, baseline 10.5±6.5; f/up 10.0±6.4 (*p* = 0.39)  Anxiety: GAD-7, baseline 9.1±6.1; f/up 8.8±5.7 (*p* =0.56)  Suicidal Ideation: 27 (34%) youth who endorsed suicidality at baseline, 22 (81%) continued to endorse suicidality at their f/up visit, and only 4 (4%) no longer endorsed suicidality at f/up | Strengths:  Prospective  Limitations:  Limited to one clinic, variability in f/up timing, short f/up, no control group | ⊕⊕⊖⊖  Low quality evidence |
| Carmichael 2021^21^  Prospective | 44 adolescents (25 AMAB, 19 AFAB)  Age 12.8-14.6 y (mean 13.6)  F/up: 12-36 m  Period: 2011-2014  Region: United Kingdom (London)    Comparator: No control group | Psychological and behavioral assessments through questionnaires (CBCL, YSR, BIS, UGDS, CGAS)    Evaluations: T0, after 12 m, after 24 m and 36 m | GnRHa mean age at start 12.8-14.6 y Duration: 31 m    GAHT | There were no changes from baseline to 12-24 m in CBCL or YSR total t-scores or for CBCL or YSR self-harm indices, nor for CBCL total t-score or self-harm index at 36 m. Most participants reported positive or a mixture of positive and negative life changes on GnRHa. | Strengths:  Prospective  Limitations:  Small sample size,  Lack of control group  f/up >12 m only for subgroup | ⊕⊕⊖⊖  Low quality evidence |
| Costa 2015^25^  Prospective | 201 adolescents with GD (AMAB AFAB?)  Age: 12-17 y  F/up: 18 m Period: 2010-2014 Region: UK (London)  Comparator: 169 healthy controls | Psychological and behavioral assessments through questionnaires (UGDS and CGAS) | Psychological support and PS with GnRHa. age at start of 13-17 y (mean = 16.48 y).  Timeline: T1: +6 m of psychological support;  T2: +12 m of psychological support ±GnRHa for eligible T3: +18 m of psychological support ±GnRHa for eligible | At baseline, GD adolescents showed poor psychosocial functioning with a CGAS mean score of 57.7 ± 12.3.  After 6 m of psychological support, GD adolescents' global functioning improved significantly (CGAS mean score: 60.7 ± 12.5;  *p* <0.01). GD adolescents receiving also GnRHa had significantly better psychosocial functioning after 12 m of GnRHa (67.4 ± 13.9) vs who received only psychological support (60.9 ± 12.2,  *p* <0.01). | Strengths:  Prospective, Timing of f/up Limitations: None | ⊕⊕⊕⊕ High quality evidence |
| De Vries 2011^26^  Prospective | 70 adolescents with GD (33 AMAB, 37 AFAB)  Age: 11.1-17 y (mean 13.65) F/up: about 3 y Period: 2000-2008 Region: Netherlands (Amsterdam)  Comparator: general population | Psychological and behavioral assessments through questionnaires (CBCL, YSR, BDI, Trait Anger and Anxiety Scale, CGAS, UGDS, BIS) | GnRHa Age 11.3-18.6 y (mean 14.75)  Duration: 2y  Evaluations: T0 before GnRHa, T1: before GAHT | Behavioral and emotional problems decreased (*p* <0.01); AFAB had more problem behavior than AMAB Depressive symptom decreased (*p* <0.01) Trait Anger and Anxiety (no significant change, Trait anger TPI  *p* = 0.503; trait anxiety STAI  *p* =0.28; AFAB had more feelings of anger and anxiety than AMAB)  Global functioning increased (*p* =0.005); AFAB had lower score than AMAB  GD did not change (*p* =0.33); AFAB had more GD than AMAB  Body Image satisfaction did not change; AFAB were more Body dissatisfied than AMAB  No adolescent withdrew from PS, and all started cross-sex hormone treatment | Strengths:  Comprehensive psychological assessment, prospective design  Limitations:  General population as control group | ⊕⊕⊕⊖ Moderate quality evidence |
| De Vries 2014^30^  Prospective | 55 adolescents (22 AMAB, 33 AFAB)  Age: 11.1-17 y  F/up: about 7 y Period: 2008-2012 Region: Netherlands (Amsterdam)  Comparator: general population | Psychological and behavioral assessments through questionnaires (UGDS, BIS, CGAS, BDI, Spielberger’s Trait Anger and Anxiety Scale, CBCL/ABCL, WHOQOL-BREF (QoL), SWLS, SHS)  Evaluations: pre-treatment; T1 during treatment on GnRHa, at initiation of CSH; T2 post-treatment: 1 y after surgery | GnRHa 11.5-18.5 y (mean 13.6 y) Duration: 2y  GAHT 13.9-19 y (mean 16.7y)  Surgery: 18.0-21.3 y (mean 19.6 y) | Significant improvements in psychological functioning and QoL; gender dysphoria and body dissatisfaction decreased significantly post-treatment; depression, anger, and anxiety reduced over time; well-being comparable to peers  Gender Dysphoria persisted through GnRHa, remitted after GAHT and surgery (T2; *p* <0.01)  Body Dissatisfaction persisted through GnRHa, remitted after GAHT and surgery (T2, *p* <0.01)  Global function improve over time ( *p* <0.01) CBCL/ABCL improve over time ( *p* <0.01)  WHOQOL-BREF, physical, psychological, social relations (QoL): similar scores than validation studies scores mean  WHOQOL Environment mean 15.47±2.06 vs validation studies scores mean 13.7±2.6 Comparison ( *p* <0.01)  The Satisfaction with life Scale and The Subjective Happiness Scale was comparable to same-age peers | Strengths: Prospective, comprehensive psychological and emotional assessments  Limitations: General population as control group | ⊕⊕⊕⊖ Moderate quality evidence |
| Fisher 2024^4^  Prospective | 36 adolescents (14 AMAB, 22 AFAB)  Age: 14.2±1.9 y  F/up: 3-12 m  Period: 2014-2020  Region: Italy (Florence)  No control group | Psychological and behavioral assessments through questionnaires (YSR, BUT, MAST, BDI, BAI)    Evaluations: T0, after 12 m, after 24 m and 36 m | GnRHa (triptorelin 3.75 mg every 28 days) Age range: 11-15 AMAB, 9-17 AFAB Duration: 3-12 m | **Significant improvement in psychological functioning, decrease in suicidality, body uneasiness, depression, and anxiety levels.**  **Hormone and physical changes observed during triptorelin treatment significantly correlated with a reduction in suicidal ideation, anxiety, and body image concerns.** | Strenghts:  Multidisciplinary approach, longitudinal tracking of both psychological and endocrinological outcomes​  Limitations:  Small sample size, limited bone health data, lack of control group for ethical reasons​ | ⊕⊕⊕⊖ Moderate quality evidence |
| Khatchadourian 2014^42^  Retrospective | 84 adolescents with GD (45 AMAB, 37 AFAB, 2 natal males undecided)  Age: 16.9 y  F/up: mean 2 y (range 0.0-11.3) Period: 1998-2011 Region: Canada (Vancouver)  Comparator: No control group | Psychological and behavioral assessments through questionnaires (UGDS, Piers-Harris Children’s Self Concept Scale, GIQA) | GnRHa (15 AMAB, 11 AFAB) Age at start: 14.7±1.9y  GAHT (39 AMAB, 24 AFAB): 17.4±1.9y | Initiation of GnRHa therapy at an earlier stage of puberty prevents the development of unwanted secondary sexual characteristics and can alleviate distress  Suicide attempts and/or emergency department visits for suicidal ideation decreased from 12% (n = 10) before the first visit to transgender clinic to 5% (n = 4) afterward. | Strengths:  None Limitations: Retrospective, small sample size, lack of control group, variability in f/up duration and treatment initiation, objective tests of psychological functioning before and after treatment were not performed systematically | ⊕⊕⊖⊖ Low quality evidence |
| Kuper 2020^43^  Prospective | 148 adolescents with GD (55 AMAB, 94 AFAB)  Age: 9-18 y (mean 14.9±2.0 y) F/up: 1 y (range 11-18m) Period: 2014-2018 Region: USA (Dallas)  Comparator: 93 GAHT only, 30 GnRH+GAHT | Psychological and behavioral assessments through questionnaires (BIS, SCARED, QIDS) | GnRHa only Age 9.8-14.9 y (n=25) | At baseline, AMAB reported greater depression and anxiety (*p* <0.01).  During the 1-y f/up youth reported decrease in body dissatisfaction (*p* <0.01), small to moderate improvements in self-report of depressive symptoms ( *p* <0.01), and small improvements in total anxiety symptoms ( *p* <0.01). No demographic or treatment-related characteristics were associated with change over time.  Lifetime and f/up rates were 81% and 39% for suicidal ideation, 16% and 4% for suicide attempt, and 52% and 18% for nonsuicidal self-injury, respectively. | Strengths:  Prospective. Control group  Limitations:  Limited data on long-term effects  Small sample size of subjects with GD receiving PS. Short f/up | ⊕⊕⊖⊖ Low quality evidence |
| Lavender 2023^44^  Retrospective | 38 adolescents out of 109  (10 AMAB, 28 AFAB)  Age: <15 y, Tanner ≥2  F/up: 1 y on GnRHa, 1 y on GAHT  Period: 2014 - 2018  Region: UK (London)    Comparator: pre-post | Psychological and behavioral assessments through questionnaires (YSR, BIS, UGDS, CBCL, SRS-2) | GnRHa range of age at start 14.01±0.81 y, duration of GnRHa before GAHT: minimum 1 y, approximately 2 y.    followed by GAHT at mean age 16.10 y. | In participants concurrently receiving psychosocial support and hormone treatment, these items improved over the time:  Dissatisfaction with primary sexual characteristics ( *p* =0.02) and gender dysphoria ( *p* =0.01)  Social motivation ( *p* =0.04)  Internalizing behaviors ( *p* =0.03)  Self-harm and Suicidality showed a general decrease    Caregivers reported a significant reduction in internalizing (*p* = 0.03) behaviors on the CBCL after GnRHa.  Other subcategories of the YSR and CBCL were within normal ranges with no significant difference (*p* > 0.05) | Strengths:  Longitudinal data, comprehensive assessments.  Limitations:  Small sample size, lack of control group, low questionnaire completion rates. Short f/up | ⊕⊕⊖⊖  Low quality evidence |
| López de Lara 2020^45^  Prospective | 23 adolescents with GD (7 AMAB; 16 AFAB)  Age: 16 y  F/up: 1 y  Period: 2018-2019  Region: Spain (Madrid)    Comparator: 30 cisgender controls (12 AFAB; 18 AMAB) | Psychological and behavioral assessments through questionnaires (UGDS, SDQ-Cas, STAI, BDI Second Edition) | GnRHa (age of start not known)  +GAHT    Range of age 14-18 y Duration: 1 y after GAHT | Significant improvement in emotional symptoms, behavior problems, hyperactivity, pro-social conduct, anxiety, and depression in the transgender group, with scores becoming similar to controls    Emotional symptoms T0: 5.2±1.6, T1: 3.4±1.2 ( *p* <0.01)  Behavior problems T0: 2.7±0.8, T1: 1.8±1 ( *p* <0.01)  Hyperactivity symptoms T0: 4±1.9, T1: 2.6±1.8 ( *p* <0.01)  Pro-social conduct T0: 8±1.6, T1: 9± 1.2 ( *p* <0.01)    Anxiety as a state T0: 33.3±9.1, T1: 16.8±8.1 ( *p* <0.01)  Anxiety as a trait T0: 33±7.2, T1: 18.5±8.4 ( *p* <0.01)    Depressive symptomatology T0: 19.3±5.5, T1: 9.7±3.9 ( *p* <0.01) | Strengths:  Prospective  Limitations:  Limited to one clinic, small sample size, only two time points analyzed. Short f/up | ⊕⊕⊖⊖  Low quality evidence |
| McGregor 2024^46^    Retrospective Cross-sectional | 438 adolescents with GD (105 AMAB, 337 AFAB)    Age: 13-17 y  F/up: no  Period: 2017-2021  Region: USA (Boston)    Comparator: 40 received GnRHa (Tanner stage 2 or 3), 398 did not receive GnRHa | Psychological and behavioral assessments through questionnaires before starting GAHT (YSR) | GnRHa (n=40) mean age at start 12 y; f/up until hormone readiness assessment | Use of GnRHa was associated with significantly lower T-scores for internalizing, anxiety, depression, stress, and total problems; fewer suicidal thoughts reported among youth who received blockade | Strengths:  Control group  Limitations:  Retrospective, cross-sectional, small sample size of GnRH treated | ⊕⊕⊖⊖  Low quality evidence |
| McPherson 2023^47^  Prospective  (Reanalysis of the study of Carmichael 2021) | 44 adolescents (25 AMAB, 19 AFAB)  Age 12.8-14.6 y (mean 13.6 y)  F/up: 3 y  Period: 2011-2014  Region: UK (London)    Comparator: USA general population (not UK) | Psychological and behavioral assessments through questionnaires (CBCL and YSR)    Evaluations: T0, after 12 m, after 24 m and 36 m | GnRHa range of age at start: 12-15 y Duration: 31 m (20-42)    GAHT | 15-34% of participants deteriorated depending on the subscale, time point and parent versus child report, and between 9% and 20% reliably improve | Strengths: None  Limitations:  Small sample size, USA control group | ⊕⊕⊖⊖  Low quality evidence |
| Russell 2021^53^  Prospective | 95 adolescents  (38 AMAB, 57 AFAB)    Age: mean 13.6 ± 0.11  F/up: 1 y  Period: n.a.  Region: UK (London)    Comparator: pre-post | Autism assessment (SRS-2) | GnRHa;  range of age at start: 9.9 to 15.9 y, mean duration: ~1 y. | No significant differences in SRS-2 scores over time and between birth assigned sex  No interactions between time and birth assigned sex were established for SRS-2 subscales or total scores. | Strengths: Prospective  Limitations:  Lack of clinical diagnosis for autism spectrum, lack of control group, short f/up | ⊕⊕⊖⊖  Low quality evidence |
| Tordoff 2022^24^  Prospective | 104 adolescents (27 AMAB, 63 AFAB, 10 NB, 4 unknown)    Age: 15.8 y  F/up: 12 m  Period: 2017-2018  Region: USA (multisite)    Comparator: hormonal treatment vs. no treatment | Surveys and medical record reviews over 12 m.  Psychological and behavioral assessments through questionnaires (PHQ-9, GAD-7) | At baseline: 1 received GnRHa only, 4 received GAHT only, 2 received both  By the end of the study: 5 received GnRHa only, 50 received GAHT only, 14 received both | Reduced depression and suicidality with GnRHa or GAHT    After adjustment for temporal trends and potential confounders, 60% lower odds of depression and 73% lower odds of suicidality among youths who had initiated GnRHa or GAHT compared with youths who had not. | Strengths: Prospective design, control group  Limitations:  Small sample size, short f/up | ⊕⊕⊕⊖ Moderate quality evidence |
| Turban 2020^56^  Cross-sectional | 89 adults with GD who received GnRHa (AMAB, AFAB)    Age: 18-36 y (23.4±5)  F/up: unavailable  Period: 1998-2015  Region: USA    Comparator: 3494 adults with GD, who wanted but did not receive GnRHa treatment | Psychological and behavioral assessments through questionnaires (Kessler Psychological Distress Scale (K6)) | GnRHa (n=89) vs not receive GnRHa (n=3405) | Subjects who received GnRHa vs not receiving, had lower odds of lifetime suicidal ideation. Adjusted odds ratio for demographic variables and level of family support for gender identity was 0.3    Suicidal ideation and suicide attempts:  -past 12 m: ideation: OR 0.6 (*p* <0.01)  -lifetime: ideation: OR 0.3 (*p* <0.01)  Mental health and substance use:  -past-month severe psychological distress: OR 0.5 (*p* <0.01) | Strengths:  Control group  Limitations:  Cross-sectional design  Missing data about the age of onset and the duration of PS | ⊕⊕⊖⊖  Low quality evidence |
| Van der Miesen 2020^29^  Retrospective cross-sectional | 178 adolescents with GD (68 AMAB, 110 AFAB)    Age: mean 16.8 y    F/up: unavailable    Period: 2012-2015    Region: Netherlands (Amsterdam)    Comparator: 272 adolescents with GD  (116 AMAB, 156 AFAB) not receiving GnRHa; age: mean 14.5 y; 651 cisgender adolescents from the general population; age: mean 15.4 y | Psychological and behavioral assessments through questionnaires (YSR) | GnRHa vs  no treatment | Before GnRHa clinic-referred adolescents showed more internalizing problems and reported increased self-harm/suicidality and poorer peer relations vs their age-equivalent peers    Adolescents receiving GnRHa had fewer emotional and behavioral problems vs cisgender peers from the general population; self-harm/suicidality was similar to cisgender peers population | Strengths:  Control group  Limitations:  Cross-sectional design, no long-term causal inference | ⊕⊕⊕⊖ Moderate quality evidence |
