# Supplementary material for: Use of gonadotropin-releasing hormone agonists in transgender and gender diverse youth: a systematic review
Source: Front Endocrinol (Lausanne). 2025 May 14;16:1555186. doi: 10.3389/fendo.2025.1555186 (PMC12116301; doi:10.3389/fendo.2025.1555186)
Supplement: Supplementary file 6 [file Table4.docx]

**Supplementary Table 1D**. Literature analysis after PICOS selection: summary of the studies and evidence grading for each study that reported fertility, sexual function, side effects and risk of cancer of GnRHa treatment. *Data are expressed as mean±SD, unless otherwise stated.*

Abbreviations: AFAB: Assigned Female At Birth; AMAB: Assigned Male At Birth; GAHT: Gender Affirming Hormone Therapy; GD: Gender Dysphoria; NS: Not Significant; PICOS: Population, Intervention, Comparison, Outcome, Study Design; PS: Pubertal Suppression; SD: Standard Deviation

| **Fertility and sexual function** | | | | | | |
| --- | --- | --- | --- | --- | --- | --- |
| **Study design** | **Sample, gender identity, age and comparator** | **Methods** | **Treatment (Range of age at start and mean duration)** | **Outcomes** | **Study limitations** | **Level of evidence** |
| De Nie 2022^38^  Retrospective | 214 AMAB (subdivided by Tanner stage at treatment initiation: Tanner stage 2-3, 4-5, adult)  Age: 29.6 ± 12.4 y  F/up: not reported  Period: 2006-2018  Region: Netherlands (Amsterdam)  No direct control group | Histological and immunohistochemical analyses of orchiectomy specimens to assess the presence and maturity of germ cells | 78 GnRHa + GAHT  136 only GAHT  Mean duration of medical treatment: 3.3 ± 2.0 y  29 subjects started medical treatment at Tanner stage 2-3,  49 at Tanner stage 4-5 and  136 as adults | In 100% of subjects who initiated medical treatment in Tanner stage 2-3 the orchiectomy specimen presented immature germ cell  **Initiation of medical treatment in early pubertal adolescents limits the ability to retrieve mature spermatozoa** that can directly be used for assisted reproductive techniques (maturation techniques in vitro seem to be necessary)  **Spermatogenesis is more negatively affected by GAHT started in adults:** complete absence of germ cells only in 15 subject (7%) who all initiated GAHT as adults  No association between duration of GAHT and the possibility for preservation of spermatozoa (OR 0.75) or spermatogonial stem cells (OR 1.03) | Strenghts:  Detailed analysis of germ cell development by Tanner stage  Limitations:  Lack of control group,  lack of serum hormone data at surgery, varying hormone formulations used. | ⊕⊕⊖⊖  Low quality evidence |
| Pang 2020^50^  Retrospective | 102 adolescents (53 AMAB, 49 AFAB)  Age 10.8 - 18.3 y F/up: not reported Period: 2003-2017  Region: Australia (Melbourne)  No direct control group | Medical record review assessing fertility counseling and preservation uptake among transgender adolescents receiving hormonal treatment | GnRHa, and GAHT; fertility preservation options assessed before hormone therapy initiation  (no details available) | **While none of AFAB pursued fertility preservation, 62% of AMAB pursued fertility preservation** (43.4% before GnRHa, 56.6% before estrogen): ⅔ froze sperm after providing a masturbatory sample (mean age 15.6± 1.4 y), ⅓ underwent testicular biopsy (mean age 13.9±1.5 y) 45% with mature sperm, 55% with germ cells only, all of which were cryopreserved | Strenghts:  Limitations:  No control group, potential selection bias, and differing access to fertility preservation options | ⊕⊕⊖⊖  Low quality evidence |
| Segev-Becker 2020^54^  Retrospective | 106 adolescents (47 AMAB, 59 AFAB)  10 pre-pubertal: (9 AMAB, 1 AFAB) age 4.3-10.5 y  96 pubertal: 38 AMAB (15.9±1.7 y), 58 AFAB (15.2±1.7 y)  F/up: not reported Period: 2013-2018 Region: Israel (Tel Aviv)  No direct control group | Review of medical records for demographic data, treatment initiation, and fertility preservation consultation | GnRHa in 77 (80%) Mean age at start 15.9±1.6 y Duration: n.a.  GAHT in 61 (64%) Age: 16.5±1.3 y  Duration: n.a. | 31% received formal fertility preservation consultation; 45% of AMAB and 6.5% of AFAB completed fertility preservation prior to hormonal treatment  Individuals who presented as transgender at younger age were more likely to undergo fertility preservation (p=0.07) | Strenghts:  Limitations:  Retrospective, lack of control group Potential referral bias, no data on fertility as an outcome, no hormonal data | ⊕⊖⊖⊖  Very low quality evidence |
| **Side effects and cancer** | | | | | | |
| Karamanis 2023^32^  Retrospective | 410 individuals (199 AMAB, 211 AFAB)  Age 17.8 y (10.7-75.6) F/up: 1.4 y (299 days-10.1 y) Period: 2006-2016  Region: Sweden  Comparator: 3,820 individuals with GD not treated with GnRHa; 73,096 individuals without GD | National registers analysis; Incidence rates of IIH calculated using Poisson distribution from Swedish national registers. F/up from first GD diagnosis or GnRHa initiation, censored at death or emigration | GnRHa; median age 17.8 y; 73% followed for ≥240 days after GnRHa initiation | **No change in incidence of intracranial hypertension** with or without GnRHa treatment  - in GD and GnRHa: n=0 (incidence rate per 100,000 per y: 0)  - in GD and no GnRHa: n=2 (incidence rate per 100,000 per y 15.7)  - in individuals without GD and without exposure to GnRHa: n=8 (incidence rate per 100,00 per y of 3.2) | Strenghts: Comparator group  Limitations:  Few cases, wide f/up range | ⊕⊕⊖⊖  Low quality evidence |
| KhatChadourian 2014^42^  Retrospective | 84 adolescents with GD (45 AMAB, 37 AFAB, 2 natal males undecided)  Age: 16.9 y  F/up: mean 2 y (range 0.0-11.3) Period: 1998-2011 Region: Canada (Vancouver)  Comparator group: pre vs post | UGDS, Piers-Harris Children’s Self Concept Scale, GIQA | GnRHa in 27 (15 AMAB, 11 AFAB) Age at start: 14.7±1.9y  GAHT in 63 (39 AMAB, 24 AFAB): 17.4±1.9y | 1 sterile abscess with leuprolide acetate (switched to triptorelin, well tolerated)  1 leg pains and headaches, resolved without treatment  1 weight gain (19 kg within 9 m), BMI was already >85th before initiation of GnRHa. | Strengths:  Limitations:  small sample size; no untreated control group Few cases (secondary outcome) Wide f/up duration, short f/up | ⊕⊖⊖⊖  Very low quality evidence |
| Perl 2020^51^  Retrospective | 19 adolescents (all AMAB); mean age at GnRHa initiation 15.7± 1.6 y  F/up: ≥2 m (9±6 m) Period: 2013-2020 Region: Israel (Tel Aviv)  Comparator: pre-post | Medical record review  BP measured every 4 m  were converted to BP percentiles for the assigned sex at birth (male)  Anthropometric, hormonal levels, smoking, alcohol consumption, drug abuse and reported anxiety diagnosis | GnRHa; age at start GnRHa 15.7±1.6 y  F/up GnRHa:  GAHT (estrogen) (n=15); age at start 18.5±1.5 y f/up estrogen: 18.5 m (range 3–63 m) | No significant changes in SBP and DBP percentiles after GnRHa and estrogen treatment | Strenghts:  Limitations:  Small sample size, no untreated control group, short F/up | ⊕⊖⊖⊖  Very low quality evidence |
| Schagen 2016^10^  Prospective | 116 adolescents (49 AMAB, 67 AFAB)  Age (median): 13.6 y (AMAB) -14.2 y (AFAB)  F/up: 3 m - 3 y  Period: 1998 - 2009  Region: Netherlands (Amsterdam)  Comparator group: pre vs post | Physical exams every 3 m  Blood tests for hormones and liver/renal function every 3-6 m  DXA for body composition | GnRHa (triptorelin 3.75 mg at 0, 2, and 4 wks, then every 4 wks)  range of age at start: AMAB 11.6-17.9 y, AFAB 11.1-18.6 y; for at least 3 m; mean duration not specified | No abnormalities in LFT or creatinine | Strenghts: Prospective  Limitations:  No control group, wide f/up range, secondary outcome. Small sample size | ⊕⊕⊖⊖  Low quality evidence |
| Segev-Becker 2020^54^  Retrospective | 106 adolescents (47 AMAB, 59 AFAB)  10 pre-pubertal: (9 AMAB, 1 AFAB) age 4.3-10.5 y  96 pubertal: 38 AMAB (15.9±1.7 y), 58 AFAB (15.2±1.7 y)  F/up: non reported  Period: 2013-2018  Region: Israel (Tel Aviv)  Comparator group: pre vs post | Review of medical records for demographic data, treatment initiation, and fertility preservation consultation | GnRHa in 77 (80%)  Mean age at start 15.9±1.6 y  Duration: n.a.  GAHT in 61 (64%)  Age: 16.5±1.3 y  Duration: n.a. | GnRHa:  - exacerbation of depression in 1 individual  - hot flashes in 7 AFAB, 2 AMAB  GAHT:  - elevated prolactin levels in 1 AMAB  - brief clitoral pain 1 in 1 AFAB | Strenghts  Limitations:  Few cases (secondary outcome). Small sample size | ⊕⊕⊖⊖  Low quality evidence |
| van Heesewijk 2023^58^  Cross-sectional | 35 prepubertal children (20 AMAB, 15 AFAB), 41 adolescents with GD (20 AMAB, 21 AFAB)  Age range for prepubertal children: 7.8–12.0 y; Age range for adolescents: 12.8–19.3 y F/up: not reported Period: not reported Region: Netherlands (Amsterdam)  Comparator: 79 cisgender peers | DTI data to measure fractional anisotropy (FA) values and white-matter microstructure | GnRHa (triptorelin monthly injections)  Mean duration 22.7 m (range 2–48 m) | Transgender adolescents showed lower FA (lower FA (indexing less longitudinal organization, fiber coherence, and myelination) in inferior fronto-occipital fasciculus, forceps major, and corpus callosum compared to cisgender peers; Negative correlation between FA values in right IFOF and cumulative GnRHa dose | Strengths:  Control group  Limitations:  Small sample, cross-sectional design, no masking in FA averaging | ⊕⊕⊖⊖  Low quality evidence |
| Walnder 2023^59^  Retrospective | 33 adolescents (10 AMAB, 23 AFAB)  Age: 9-18 y F/up: not reported Period: 2018- 2019 Region: Canada (Alberta)  Comparator: pre-post | Retrospective analysis of electronic medical records, including ECG results and medication use; QTc intervals calculated using the Bazett formula | GnRHa (monthly or 3-monthly)  Age 13.7±2.1 y  12 GnRHa + GAHT (7 testosterone - 5 estradiol) | No QTc prolongation >460 ms observed; borderline QTc prolongation in 24.2% of youth; no significant difference in QTc interval from baseline | Strengths  Limitations:  Small sample size, no untreated control group, lack of baseline ECG for some participants | ⊕⊖⊖⊖  Very low quality evidence |
